# Retinoic acid signaling is critical during the totipotency window in early mammalian development

**DOI:** 10.1038/s41594-021-00590-w

**Published:** 2021-05-27

**Authors:** Ane Iturbide, Mayra L. Ruiz Tejada Segura, Camille Noll, Kenji Schorpp, Ina Rothenaigner, Elias R. Ruiz-Morales, Gabriele Lubatti, Ahmed Agami, Kamyar Hadian, Antonio Scialdone, Maria-Elena Torres-Padilla

**Affiliations:** 1 Institute of Epigenetics and Stem Cells (IES), Helmholtz Zentrum München, Munich, Germany; 2 Institute of Functional Epigenetics (IFE), Helmholtz Zentrum München, Neuherberg, Germany; 3 Institute of Computational Biology (ICB), Helmholtz Zentrum München, Neuherberg, Germany; 4 Assay Development & Screening Platform, Institute of Molecular Toxicology & Pharmacology (TOXI), Helmholtz Zentrum München, Neuherberg, Germany; 5 Wellcome Sanger Institute, Wellcome Genome Campus, Hinxton, Cambridge, UK; 6 Faculty of Biology, Ludwig-Maximilians Universität, Munich, Germany

**Keywords:** Molecular biology, Developmental biology

## Abstract

Totipotent cells hold enormous potential for regenerative medicine. Thus, the development of cellular models recapitulating totipotent-like features is of paramount importance. Cells resembling the totipotent cells of early embryos arise spontaneously in mouse embryonic stem (ES) cell cultures. Such ‘2-cell-like-cells’ (2CLCs) recapitulate 2-cell-stage features and display expanded cell potential. Here, we used 2CLCs to perform a small-molecule screen to identify new pathways regulating the 2-cell-stage program. We identified retinoids as robust inducers of 2CLCs and the retinoic acid (RA)-signaling pathway as a key component of the regulatory circuitry of totipotent cells in embryos. Using single-cell RNA-seq, we reveal the transcriptional dynamics of 2CLC reprogramming and show that ES cells undergo distinct cellular trajectories in response to RA. Importantly, endogenous RA activity in early embryos is essential for zygotic genome activation and developmental progression. Overall, our data shed light on the gene regulatory networks controlling cellular plasticity and the totipotency program.

## Abstract (web summary)

High-throughput chemical screening identifies retinoic acid signaling as a regulatory pathway of 2-cell-like cell reprogramming and early mouse development.

## Main

Totipotency is the ability of a cell to give rise to a full organism^1,2^ and encompasses the broadest cellular plasticity in the mammalian body. Totipotency is a transient feature of the cells in the early embryo, which in mice is limited to the zygote and 2-cell embryo, because only the blastomeres of these stages can autonomously generate a full organism^3–5^. As development progresses, totipotency is lost and cellular plasticity is gradually reduced. Three days after fertilization, the blastocyst forms and pluripotent cells emerge within the inner cell mass (ICM)^2^. In contrast to totipotent cells, pluripotent cells can no longer contribute to the extra-embryonic derivatives of the trophectoderm^6^.

Pluripotent embryonic stem (ES) cells derive from the ICM. The establishment of ES cell lines over 30 years ago^7^ has enabled their use as model system to study pluripotency. Depending on the culture conditions, ES cell cultures can be highly heterogeneous, in which distinct cell populations with diverse developmental potentials coexist. Among these, cells resembling the blastomeres of 2-cell stage embryos, referred to as ‘2-cell-like-cells’ (2CLCs), arise spontaneously, constituting less than 1% of the cells^8^. 2CLCs share several features with 2-cell stage embryos, including a ‘2 C’ transcriptional program, characterized by genes expressed upon zygotic genome activation (ZGA), which occurs in late 2-cell embryos^8–10^. This includes the transcription factor ZSCAN4^11^ and retrotransposons from the MERVL family^12^. In addition, 2CLCs recapitulate other features of 2-cell embryos including their chromatin accessibility landscape^9^, greater global histone mobility^13^ and the capacity to contribute to extra-embryonic tissues^8^.

Although not strictly totipotent, 2CLCs are considered totipotent-like cells and are therefore a powerful cellular model to study molecular features related to totipotency. 2CLCs emerge most often from naive ES cells, but downregulate protein levels of pluripotency factors^10^. Upon exit from pluripotency, 2CLCs arise from an intermediate cellular population characterized by the expression of ZSCAN4. The number of ZSCAN4^+^ cells fluctuates in cell cultures, and can increase following changes in metabolites in the medium or the addition of signaling molecules such as retinoic acid (RA)^14,15^. Much effort has been made towards understanding the mechanisms regulating the transcriptional program in 2CLCs and in 2-cell stage embryos^8–10,16–21^. However, it is still unclear how 2CLCs arise, and the factors that activate the 2-cell program and regulate ZGA in vivo remain elusive. Thus, identifying conditions that can robustly induce and stably maintain 2CLCs in culture can shed light into their regulatory networks and potentially uncover key factors activating the earliest developmental program in mammals.

## Results

### Low concentrations of RA induce 2CLCs

To identify the molecular pathways underlying 2CLC identity, we performed a large-scale, small-molecule screen using an ES cell line with a stable integration of the ‘*2C::tbGFP*’ reporter, driving turbo GFP expression under MERVL long-terminal repeat (LTR; Supplementary Fig. 1a), used to identify 2CLCs^8–10,16,17^. We set up a pilot screen with 1,280 FDA-approved compounds using the percentage of tbGFP-expressing cells as primary readout. As a positive control for 2CLC induction we used acetate^14^. Our pilot set-up performed robustly across experiments (Supplementary Fig. 1b–d). We then screened 30,000 compounds from a diversity library and obtained 393 hits (Supplementary Fig. 1b), which we further assayed in triplicates and under two concentrations, incorporating ZSCAN4 expression as additional readout. This resulted in 16 confirmed hits, which we tested in a tertiary screen using a concentration gradient and a viability test. In general, higher concentrations of these 16 hits led to reduced cell numbers (Supplementary Fig. 1e), suggesting dose-dependent toxicity. The tertiary screen identified three retinoids as major hits for their ability to increase the number of 2CLCs: RA, isotretinoin and acitretin (Supplementary Fig. 2a,b). Because RA is the only natural retinoid among them, we focused primarily on RA for further studies. We validated the screening using fluorescence-activated cell sorting (FACS), which confirmed that RA induces 2CLCs, with an effect size of ~10-fold (Supplementary Fig. 2c).

**Fig. 1:**
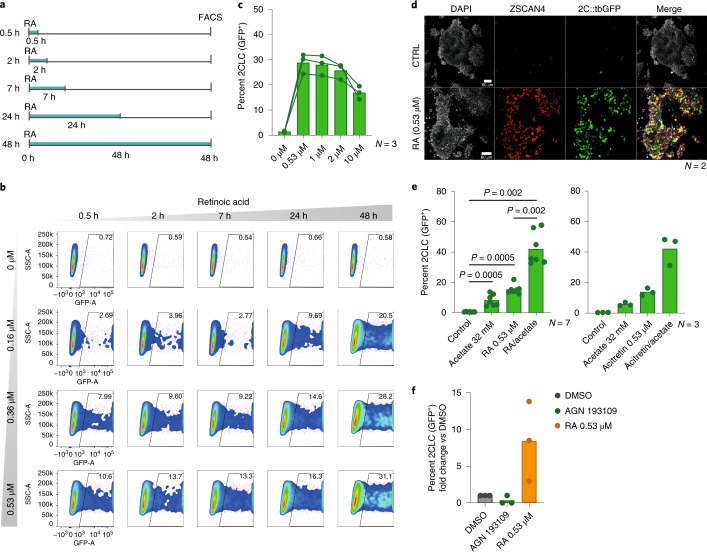
Low concentrations of RA robustly induce 2CLCs. **a**, Experimental design. Embryonic stem (ES) cells were treated with a range of RA concentrations for different time periods. 2CLC induction was measured by FACS, 48 h after treatment. **b**, Representative scatter plot for the experiment in **a**, showing *2C::tbGFP* fluorescence measurements of individual cells as assayed by FACS. **c**, Effect of high RA concentrations on 2CLCs induction. The percentage of 2CLCs (GFP^+^) quantified by FACS 48 h after treatment is shown (bars show the mean of the indicated number of replicates). Each line and connecting dots correspond to measurements of one replicate. **d**, Immunofluorescence using antibodies for the indicated proteins. The merge images show 4′,6-diamidino-2-phenylindole (DAPI; gray), ZSCAN4 (red) and tbGFP (green) expression. Scale bars, 80 µm. **e**, Effect of treatment with retinoids in combination with acetate on 2CLC induction. The percentage of 2CLCs (GFP^+^) was quantified by FACS, 48 h after treatment. The mean of the indicated replicates (represented by individual dots) is shown. *P* values were calculated by two-sided Mann–Whitney test. **f**, Induction of 2CLCs from ZSCAN4^+^ cells upon RA treatment. The percentage of 2CLCs (GFP^+^/mCherry^+^) was quantified by FACS, 24 h after sorting ZSCAN4^+^ (GFP^−^/mCherry^+^) cells.

Next, we characterized the conditions that allow robust reprogramming to 2CLCs by RA. We also aimed to reduce the DMSO concentration because DMSO hampers 2CLC emergence (Supplementary Fig. 2c). Because, in our screen, we observed 2CLC induction at the lowest RA doses, we probed these RA concentrations with reduced DMSO concentrations and different treatment lengths (Fig. 1a). Remarkably, we identified conditions under which RA induced a more than 50-fold increase of 2CLCs (up to 30% of the culture; Fig. 1b). Although we observed an increase in 2CLC induction with higher RA concentration and length of treatment, just 30 min of RA treatment at the lowest concentration (0.16 µM) robustly increased (approximately fourfold) 2CLCs (Fig. 1b). We obtained similar results, albeit with slightly lower induction rates, for the other retinoid, acitretin (Supplementary Fig. 3a).

RA has been used for decades to induce ES cell differentiation^22^, which appears at odds with its ability to induce 2CLCs. However, RA induces differentiation at higher doses (1–10 µM) than those we report here to induce 2CLCs, and when added for longer time periods. Indeed, increasing the RA concentration (up to 10 µM) did not lead to a higher proportion of 2CLCs (Fig. 1c). Instead, we observed maximal 2CLC induction at 0.53 µM RA, and higher concentrations gradually decreased this effect (Fig. 1c). Thus, RA mediates 2CLC reprogramming most efficiently at lower concentrations. 2CLCs induced with RA express 2CLC markers such as ZSCAN4 (Fig. 1d). The simultaneous addition of RA or acitretin with acetate—also known to induce 2CLCs^14^—resulted in a synergistic effect, leading to a conversion of more than 40% of the ES population into 2CLCs (Fig. 1e and Supplementary Fig. 3b). We next addressed whether RA plays a role in the transition from ZSCAN4^+^ cells to 2CLCs. We used a double ‘2C’ and *Zscan4* reporter cell line^10^, sorted *Zscan4*^*+*^*/2C::tbGFP*^*−*^ cells, and treated them with RA. RA treatment increased the number of 2CLCs arising from ZSCAN4^+^ cells (Fig. 1f), and induction of 2CLCs from ZSCAN4^+^ cells was blocked by an antagonist of RA signaling (Fig. 1f). These data indicate that RA promotes the transition to the 2CLC state from the intermediary ZSCAN4^+^ cell population. Thus, we conclude that low doses of RA robustly induce 2CLC reprogramming.

### The RA pathway is active in spontaneously emerging 2CLCs

**Fig. 2:**
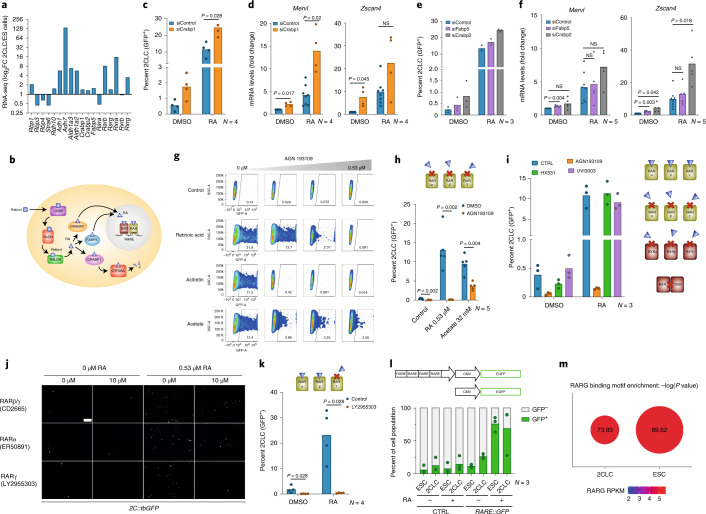
RARγ is required for 2CLC emergence. **a**, Expression levels (log_2_FC) (FC, fold change) of selected RA-pathway-related genes in 2CLCs and ES cells (ESCs) based on RNA-seq data (*N* = 2, from ref. ^16^). **b**, Schematic of the RA pathway. **c**, Induction of 2CLCs upon siRNA for *Crabp1* and RA treatment. The percentage of 2CLCs was quantified by FACS. The mean ± s.d. of the indicated number of replicates is shown. *P* values were calculated by two-sided Mann–Whitney test. **d**, Quantitative polymerase chain reaction (qPCR) analysis upon transfection of siRNA for *Crabp1* and RA treatment. Mean ± s.d. values of the indicated number of replicates are shown. *P* values were calculated by two-sided Student’s *t*-test. NS, not significant. **e**, Induction of 2CLCs upon transfection of siRNA for *Fabp5* and *Crabp2* and RA treatment. The percentage of 2CLCs was quantified by FACS. The mean ± s.d. of the indicated number of replicates is shown. **f**, qPCR analysis after transfection of siRNA for *Fabp5* and *Crabp2* and RA treatment. Mean ± s.d. values of the indicated number of replicates are shown. *P* values were calculated by two-sided Student’s *t*-test. **g**, Representative scatter plots from data in 3 h showing *2C::tbGFP* fluorescence measurements of individual cells as assayed by FACS. **h**, Induction of 2CLCs upon treatment with AGN193109. The percentage of 2CLCs was quantified by FACS, 48 h after treatment. Mean values of the indicated replicates are shown. *P* values were calculated by two-sided Mann–Whitney test. **i**, Induction of 2CLCs upon treatment with RAR and RXR antagonists. The percentage of 2CLCs was quantified by FACS, 48 h after treatment. Mean ± s.d. values of the indicated replicates are shown. **j**, Representative fluorescence images of ES cell colonies harboring the *2C::tbGFP* reporter, 48 h after treatment with the indicated antagonists and RA. Scale bar, 100 µm. **k**, Induction of 2CLCs upon treatment with LY2955303. The percentage of 2CLCs was quantified by FACS, 48 h after treatment. The mean of the indicated replicates is shown. *P* values were calculated by two-sided Mann–Whitney test. **l**, Percentage of 2CLCs displaying RARE activity. The percentage of 2CLCs (tdTOMATO^+^) and ES cells (tdTOMATO^−^) with RARE activity (GFP^+^) was quantified by FACS, 48 h after *RARE::EGFP* reporter transfection and 24 h after RA treatment. The mean of the indicated replicates is shown. **m**, RARγ binding motif enrichment in open chromatin regions, using 2CLC and ES cell specific peaks. Dot size: −log_10_(*P* value).

We next explored whether RA signaling is responsible for the spontaneous emergence of 2CLCs. Analysis of 2CLC RNA-seq datasets^16^ revealed an increase in the expression of some of the genes encoding proteins mediating the conversion of retinol to RA, such as RDH10 and ALDH1A2 and ALDH1A3^23^. The nuclear receptors RAR (retinoic acid receptor) and RXR (retinoid X receptor) also showed increased expression in 2CLCs (Fig. 2a). This suggests that the RA pathway might be active in 2CLCs, and possibly also in totipotent cells in vivo.

To investigate the mechanism whereby RA induces 2CLCs, we disrupted the RA signaling and degradation pathways. First, we disrupted cellular RA metabolism by perturbing RA degradation through the downregulation of CRABP1, which mediates RA clearance (Fig. 2b)^24^. siRNA for *Crabp1* increased 2CLC induction in response to RA (Fig. 2c and Supplementary Fig. 4a) and led to a strong upregulation of *Zscan4* and endogenous *Mervl* transcripts (Fig. 2d). Importantly, *Crabp1* downregulation also increased the 2CLC population in control conditions (Fig. 2c), indicating that the RA pathway might be involved in triggering spontaneous reprogramming of 2CLCs. Second, we addressed whether 2CLC induction relies on nuclear RA function. We performed siRNA against the RA importers CRABP2 and FABP5, which bind RA and translocate into the nucleus to facilitate RA binding to RAR or PPAR, respectively, enabling transcriptional activation of RA-response genes^24^ (Fig. 2b). Downregulation of *Crabp2* or *Fabp5* did not prevent 2CLC induction and resulted instead in a small, reproducible increase in RA-mediated 2CLC reprogramming (Fig. 2e). We observed similar results, albeit not significant, without RA addition (Fig. 2e). The slight increase in 2CLC was accompanied by an increase in *Zscan4* and *Mervl* expression (Fig. 2f). Because altering the levels of the nuclear RA importers affects 2CLC number, these results suggest that the RA pool in the nucleus plays a role in 2CLC induction.

### The transcription factor RARγ mediates 2CLC reprogramming

We next addressed whether 2CLCs depend on downstream transcriptional activity of RA. Following RA import into the nucleus, RA binds to RARs and RXRs^25^. In the canonical pathway, these receptors form heterodimers upon ligand binding and activate transcription of targets containing retinoic acid response elements (RAREs). RXRs can also form non-canonical heterodimers with other nuclear receptors^26^. Thus, we tested whether specific transcription factors are necessary for RA-induced 2CLC reprogramming. We first asked whether 2CLC induction by RA and acitretin is affected by a general RAR antagonist, AGN193109^27,28^. AGN193109 clearly blocked 2CLC induction by RA and acitretin (Fig. 2g,h), indicating that 2CLC reprogramming upon retinoid stimulation depends on RAR activity. Interestingly, AGN193109 also reduced the effect of acetate on 2CLCs (Fig. 2g,h), suggesting that 2CLC induction by acetate is mediated partly through RAR activity. Importantly, addition of AGN193109 led to a significant reduction of the endogenous 2CLCs in control conditions, leading to a practically undetectable 2CLC population (Fig. 2g,h). Consistently, AGN193109 abolished the effect of *Crabp1*, *Crabp2* and *Fabp5* siRNA on 2CLC induction in control conditions and upon RA stimulation (Supplementary Fig. 4b). These results indicate that RAR activity mediates endogenous and RA-induced 2CLC reprogramming, pointing towards a key role for the RA pathway and its receptors in the core 2CLC network.

We next investigated whether RA activity signals through RAR homodimers or RAR/RXR heterodimers by treating ES cells with RXR antagonists in combination with RA. In contrast to the RAR antagonist (AGN193109), neither of the RXR antagonists tested affected 2CLC induction (Fig. 2i), suggesting that a non-canonical RAR dimer mediates RA activity during 2CLC induction. Because AGN193109 inhibits all RAR subtypes (α, β and γ), we next determined which RAR subtype is necessary for 2CLC induction. Inhibiting RARα and RARβ decreased RA-mediated 2CLC induction slightly, but did not abolish it (Fig. 2j). However, blocking RARγ with LY2955303 had the strongest effect in inhibiting 2CLC emergence, with an almost complete disappearance of detectable 2CLCs in control conditions, and a dramatic reduction upon RA stimulation (Fig. 2j,k and Supplementary Fig. 4c). Accordingly, RARγ participates in 2CLC induction by RA and in the spontaneous emergence of 2CLCs.

To test whether RA can activate transcription in 2CLCs, we used a RARE reporter, whereby a minimal promoter (cytomegalovirus, CMV) and an upstream RARE^29^ drive GFP expression (Fig. 2l), which we transfected into a *2C::tdTomato* ES cell line^16^. RARE reporter activity increased upon RA addition compared to the control plasmid containing the minimal promoter alone. In addition, the 2CLC population (tdTOMATO^+^) contains GFP^+^ cells (~25% of the cells; Fig. 2l). Altogether, this indicates that endogenous 2CLCs possess RARE activity and that the fraction of 2CLCs showing this activity increases upon RA stimulation. To investigate this further, we asked whether genes expressed in 2CLCs contain RARE motifs by examining 2CLC-regulatory regions from assay for transposase-accessible chromatin sequencing (ATAC-seq) datasets^30^. The RARE motif was significantly enriched in 2CLCs compared to a random distribution, which appeared both in the ‘gained’ and ‘lost’ peaks compared to ES cells (Fig. 2m). The RARE motif in 2CLC-specific peaks was also significantly enriched compared to ATAC-seq peaks shared between 2CLCs and ES cells (*P* = 1.14 × 10^−95^). We obtained similar results in ES cell-specific peaks (*P* = 1.05 × 10^−132^). Thus, enrichment of the RARE motif in accessible regions in 2CLCs correlates with the RARE activity observed in 2CLCs and suggests that RA activity functions through the binding of RARE elements in ES cells to induce 2CLC reprogramming.

### RA induces 2CLC reprogramming without inducing differentiation

**Fig. 3:**
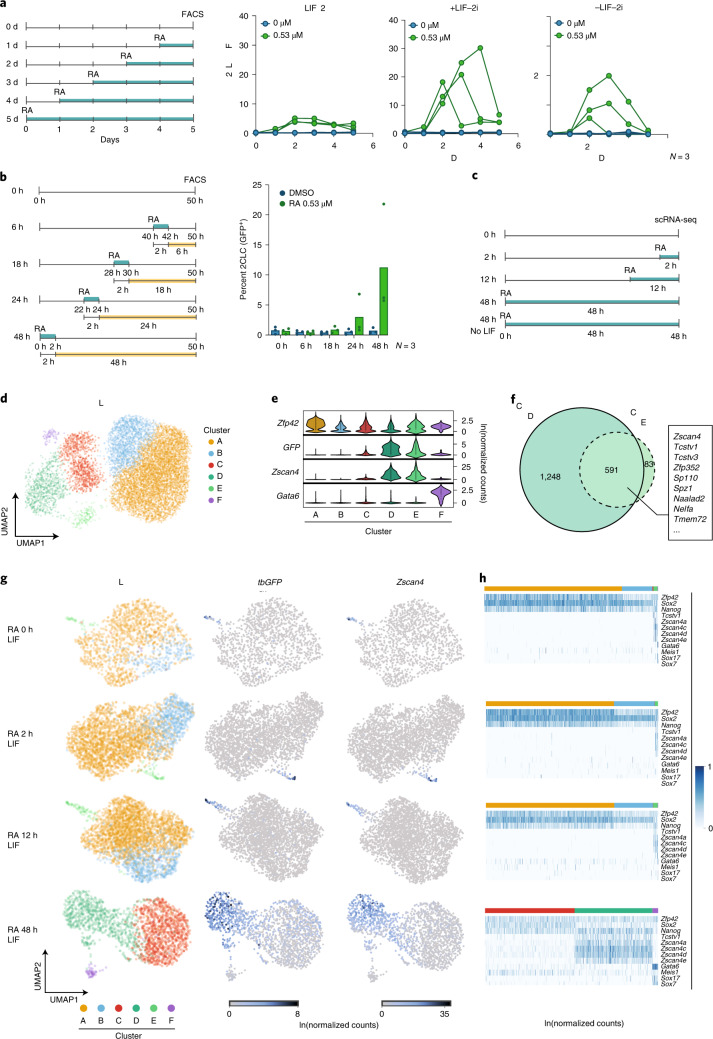
2CLC induction by RA is time-regulated and captured by scRNA-seq. **a**, Left: experimental design. ES cells containing the *2C::tbGFP* reporter were treated for a range of time periods with RA under the indicated culture conditions. 2CLC (GFP^+^) induction was measured for all samples at the same end point by FACS. Right: percentage of 2CLCs (GFP^+^) determined by FACS. Each line with connected dots corresponds to the measurement of one replicate. **b**, Left: experimental design. ES cells containing the *2C::tbGFP* reporter were treated with RA for 2 h, and the emergence of 2CLCs was measured at different timepoints after treatment. Right: percentage of 2CLCs (GFP^+^) quantified by FACS. The mean of the indicated replicates (represented by individual dots) is shown. **c**, Experimental design for scRNA-seq. ES cells containing *2C::tbGFP* reporter were treated with RA for different time periods. **d**, UMAP plot from scRNA-seq comprising all cells grown with serum/LIF and treated with RA for 0 h, 2 h, 12 h or 48 h. Cells are colored based on the clusters identified by the Leiden algorithm. **e**, Violin plots showing the expression levels of selected marker genes (rows) in each cluster (columns): *Zfp42*/*Rex1*, marker of naive ES cells (corresponding to cluster A); *Zscan4* (computed as the sum of expression counts of genes in the *Zscan4* family) and *tbGFP* (MERVL) marking 2CLCs (clusters D and E); *Gata6* for differentiating cells (cluster F). **f**, Venn diagram comparing upregulated genes in cluster D and cluster E. **g**, UMAP plots depicting scRNA-seq data from cells grown in LIF and RA for different periods of time (rows) and colored by cluster (left column), by expression level of *GFP* (MERVL) (central column) and by expression level of *Zscan4* (calculated as the sum of the levels of genes from the *Zscan4* family; right column). **h**, Heatmaps displaying the expression levels of selected marker genes in cells at different times after RA treatment as in **g** (0 h, 2 h, 12 h, 48 h). *Zfp42*/*Rex1* is a marker of naive ES cells; *Sox2* and *Nanog* mark ES cells; *Tcstv1*, *Zscan4a*, *Zscan4c*, *Zscan4d* and *Zscan4e* are upregulated in 2CLCs; *Gata6*, *Sox17* and *Sox7* display higher expression levels in differentiating cells.

2CLCs arise preferentially from naive ES cells^10^. Because RA promotes ES cell differentiation^22^, we next addressed whether the ability of RA to reprogram 2CLCs depends on culture conditions. We tested conditions that promote (1) naive, ground-state pluripotency (+LIF (leukemia inhibitory factor) and +2i), (2) primed pluripotency (+LIF without 2i) or (3) exit of pluripotency towards differentiation (withdrawal of LIF and 2i). We treated ES cells with RA for one to five days and quantified 2CLCs (Fig. 3a). For the three conditions analyzed, 2CLC induction was highest 48 or 72 h following RA addition, beyond which timepoint the 2CLC population gradually decreased (Fig. 3a). Although the addition of 2i decreased the number of RA-induced 2CLCs, LIF removal also led to a decrease in the percentage of 2CLCs (Fig. 3a). Of the three conditions, the highest reprogramming efficiency by RA was observed when LIF was maintained, but 2i was removed (Fig. 3a). These data suggest that a constant pool of pluripotent cells is required for 2CLC reprogramming upon RA addition and that, upon longer treatment, ES cells start to differentiate and are no longer able to transition towards the 2CLC state. Next, we determined the time it takes for ES cells to reprogram into 2CLCs in response to RA by adding RA to the medium for only 2 h and analyzing the percentage of 2CLCs at several timepoints thereafter (Fig. 3b). We first detected 2CLC induction 18 h after treatment and maximal induction 48 h after RA removal, suggesting that short exposure to RA induces reprogramming a few hours after the pulse. Overall, a short RA treatment is sufficient to robustly induce 2CLCs and RA may be important early during the reprogramming process.

The above results indicate that low RA concentrations robustly induce 2CLC reprogramming under a defined temporal window. To better understand how RA induces 2CLCs, we performed single cell (sc) RNA-seq at 0, 2, 12 and 48 h of RA treatment (Fig. 3c). We also analyzed cells cultured under identical RA conditions, but in the absence of LIF, as a reference for cells undergoing differentiation^31^ (Fig. 3c). We sequenced 14,742 cells across timepoints, of which 11,432 passed stringent quality criteria (Supplementary Fig. 5a,b). Clustering all data points cultured with RA and LIF revealed six clusters, visualized using uniform manifold approximation and projection (UMAP; Fig. 3d). These clusters (A–F) corresponded roughly to (A) cells with high expression levels of pluripotency factors (*Rex1/Zfp42*, *Sox2*, *Nanog*); (B) cells with a more intermediate expression level of pluripotency factors, presumably exiting pluripotency; (C) a cluster of ‘RA-responsive’ cells exclusively present in the 48 h RA treatment, which express low levels of 2CLC markers such as *Zscan4a*,*c*,*d*,*e* and *Gm47924*; (D) and (E) cells expressing 2CLC markers, such as *Zscan4a*,*c*,*d*,*e*, *Gm47924* and *Tcstv1*; (F) cells expressing early differentiation markers (*Gata6*, *Sox17*, *Sox7*) (Fig. 3e–h and Supplementary Fig. 5c). The transcriptional differences between the clusters extended beyond the known 2CLC and pluripotency markers (Supplementary Fig. 5c and Supplementary Table 1).

We analyzed each timepoint individually based on the six clusters identified, which comprise all cellular heterogeneity across timepoints. To assess whether any cluster represents the 2CLC population, we plotted *2C::tbGFP* and *Zscan4* expression over the UMAP (Fig. 3g). Both *tbGFP* and *Zscan4* were expressed highest in clusters D and E in all timepoints, indicating that unbiased clustering identifies 2CLCs based on transcriptional data (Fig. 3e). In agreement with our observations above, the number of 2CLCs (GFP^+^ cells) was maximal in the 48 h RA-treated timepoint, reaching up to 60% of the population (Fig. 3g,h and Supplementary Fig. 5d). Accordingly, *Zscan4*^*+*^ cells represented almost 80% of the cells captured at this timepoint (Supplementary Fig. 5e).

Differential gene expression (DE) analysis between clusters revealed the ‘2C’ signature in clusters D and E (Fig. 3h, Supplementary Fig. 5c and Supplementary Tables 2–7), which contained genes expressed in 2-cell embryos, including *Zscan4*, *Tcstv1* *and* *Gm20767*. The gene signature specific to cluster D overlapped significantly with that of cluster E (Fig. 3f; Fisher’s exact test *P* < 2.2 × 10^−16^). This indicates that endogenous 2CLCs (cluster E, already detected in early timepoints), overall, share the transcriptional profile of RA-induced 2CLCs (cluster D, upon induction at 48 h), including expression of *Dux* (Supplementary Fig. 5f). We also identified new 2CLC markers (Supplementary Tables 2–7), such as *Tmem72*, a transmembrane protein of unknown function (Supplementary Fig. 6a,b). The RA-responsive cluster (cluster C) emerging at 48 h displayed a partial ‘2C’ signature too (Supplementary Fig. 6c). This includes expression of *2C::tbGFP* and *Zscan4a,c,d,e*, albeit at low levels, as well as *Tcstv1* and *Gm47924* (Fig. 3e and Supplementary Fig. 5c).

**Fig. 4:**
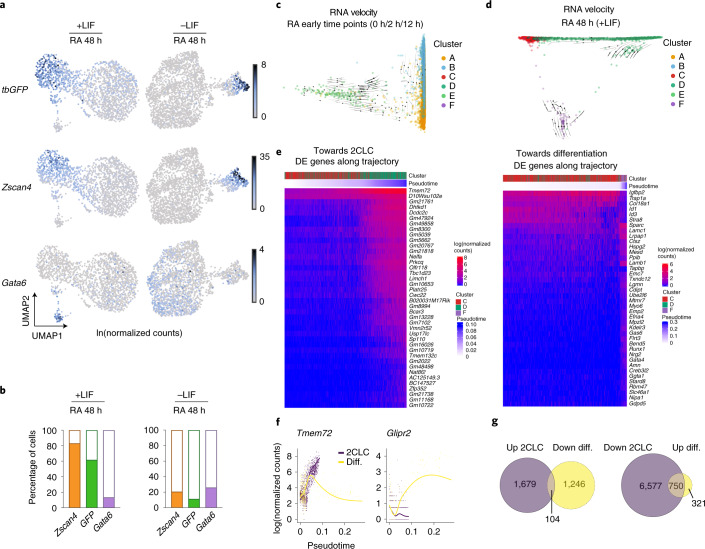
RA-reprogrammed 2CLCs differ from differentiating cells. **a**, UMAP plots of cells treated with RA for 48 h with LIF (left column) or without LIF (right column). Rows from top to bottom are colored by expression of *tbGFP* (MERVL), *Zscan4* (marking 2CLCs) and *Gata6* (marking differentiating cells). **b**, Percentages of cells where the indicated marker gene is detected (counts > 0). The left barplots refer to cells grown with LIF and the right barplots to cells grown without LIF; in both cases, cells were treated with RA for 48 h. **c**, Diffusion map with RNA velocity overlaid for cells grown in LIF and treated with RA for 0 h, 2 h and 12 h. The RNA velocity vectors indicate that cells from the ES cell clusters (A and B) are transitioning into the 2CLC cluster (E). **d**, Diffusion map with RNA velocity overlaid for cells grown in LIF and treated with RA for 48 h. Here, 2CLCs (clusters C and D) and differentiating cells (cluster F) lie on different transcriptional trajectories. **e**, Heatmaps displaying the expression of DE genes along the trajectories towards 2CLCs and towards cell differentiation based on the 48 h scRNA-seq timepoint. The cell clusters (as in Fig. 3e) and pseudotime values are indicated. **f**, Expression levels of *Tmem72* and *Glipr2* genes plotted according to the pseudotime along the cellular trajectories towards differentiation (yellow line) or 2CLCs (purple line). **g**, Venn diagram of DE genes within each of the two trajectories.

**Fig. 5:**
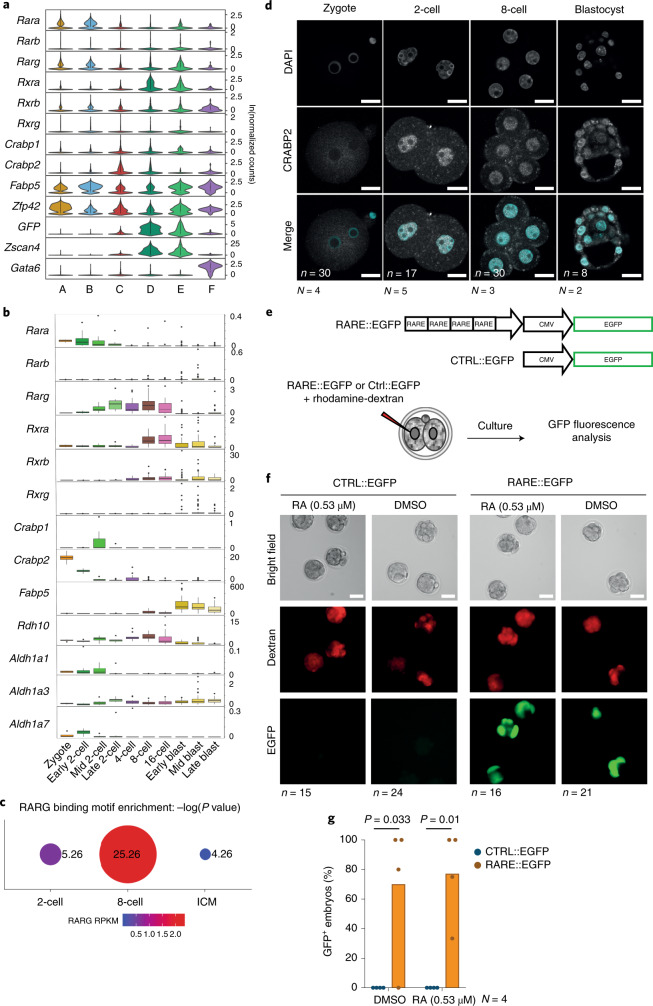
The RA pathway is active in totipotent cells of the mouse embryo. **a**, Violin plots showing the distribution of expression of RA receptors per cluster. The lower four genes are markers for naive ES cells (*Zfp42*; cluster A); 2CLCs (*Zscan4* and *tbGFP*; clusters C, D and E); and differentiating cells (*Gata6*; cluster F). **b**, Box plots depicting the expression level of the indicated RA-pathway-related genes in pre-implantation embryos at zygote (*n* = 4), early 2-cell (*n* = 8), mid 2-cell (*n* = 12), late 2-cell (*n* = 10), 4-cell (*n* = 14), 8-cell (*n* = 28), 16-cell (*n* = 50), early blastocyst (*n* = 43), mid blastocyst (*n* = 60) and late blastocyst (*n* = 30) stages. The boxes denote the 25th and 75th percentiles (bottom and top of box) and median values (horizontal band inside box). The whiskers indicate the values observed within up to 1.5 times the interquartile range above and below the box. **c**, RARG motif enrichment in the open chromatin regions of the ±10 kb TSS by indicated developmental stage. Dot size, −log_10_(*P* value). **d**, Immunostaining of CRABP2 at the indicated developmental stages. Images are single confocal sections of single embryos. *n*, number of embryos analyzed. *N*, number of experimental replicates. Scale bars, 20 µm. **e**, Experimental design for the data in Fig. 6f,g. A RARE::EGFP reporter or a control plasmid lacking the *RARE* motifs was injected in one random blastomere of 2-cell-stage embryos. **f**, Representative fluorescence images of embryos with the *RARE::EGFP* reporter 44 h after microinjection of the reporter with or without RA treatment, showing embryos between late 8-cell and cavitating morula. **g**, Percentage of embryos expressing GFP from the control (CTRL) or RARE reporter. Median values of the indicated replicates (represented by individual dots) are shown. *P* values were calculated by one-sided Mann–Whitney test.

In addition to the 2CLC clusters, the two clusters comprising pluripotent ES cells exhibiting high and medium levels of *Rex1* and *Nanog* (clusters A and B) were consistently present across early timepoints (0, 2 and 12 h) and represented the majority of the cells at these timepoints (Fig. 3g). Specifically, at time 0 h, the two largest clusters expressed pluripotency markers, while the 2CLC cluster exhibited lower expression of pluripotency genes (Fig. 3h), as expected^8,10^. With longer timepoints with RA exposure, pluripotency markers expression decreased and, by 48 h, the number of 2CLCs increased drastically and a cluster of cells expressing differentiation markers emerged (cluster F; Fig. 3g,h). Importantly, the 2CLCs and the differentiating cluster do not share expression patterns and are clearly distinguishable from each other (Fig. 3g,h). This was further demonstrated when comparing scRNA-seq profiles of cells grown for 48 h with RA with LIF and without LIF (Fig. 4a). LIF removal resulted in a larger population of cells undergoing differentiation, visible as a cluster of cells expressing markers like *Gata6* (Fig. 4a,b). In line with our results above, LIF removal resulted in fewer 2CLCs compared to cells grown in LIF, upon RA stimulation (Fig. 4b). Importantly, the 2CLC cell population (*tbGFP*^*+*^ and *Zscan4*^*+*^) did not overlap with the population of differentiating precursor cells (*Gata6*^*+*^) under these conditions either (Fig. 4a). We note that another feature that distinguishes 2CLCs (clusters D and E) from differentiating cells (cluster F) is the expression of some RA-signaling components, such as *Rxra*, which display higher expression levels in 2CLCs (see below and Fig. 5a). Thus, cells differentiating upon RA addition constitute a distinct population from 2CLCs, and ES cells can respond differently to RA stimulation, thereby generating different populations and potential cell trajectories.

To address whether RA elicits different cellular trajectories we performed RNA velocity analysis^32^. We first asked whether the scRNA-seq transcriptional dynamics faithfully recapitulates the origin of the 2CLCs that emerge from ES cells^8,10^. RNA velocity on all early timepoints (0, 2 and 12 h of RA treatment) revealed indeed a directional flow emerging from ES cells (Fig. 4c). In addition, we observed arrows denoting flow between clusters A and B, suggestive of fate transitions between naive (*Nanog/Rex1*-high) and more primed (*Nanog/Rex1*-low) ES cells, as expected^33,34^. We asked if trajectories for 2CLCs versus differentiation in response to RA can be distinguished based on transcriptional dynamics. We applied RNA velocity to our later timepoint, which revealed a strong separation between the path of differentiating precursors (purple, cluster F) and that of 2CLCs (green, cluster D) (Fig. 4d). Thus, 2CLCs undertake a clearly distinct trajectory to that of early differentiating precursors.

Next, we explored potential reasons why cells may undertake these two different trajectories. We used Slingshot to map the trajectory depicting the transition towards 2CLCs (cluster D) and the trajectory towards differentiation (cluster F) across the late timepoint. We then asked whether genes are differentially expressed along each trajectory. Different genes become activated during each transition, displaying either a sharp or a more gradual increase in gene expression (Fig. 4e,f). Among these, *Gsk3b* is downregulated in the 2CLC trajectory, suggesting potential differences in Wnt signaling underlying the differential response to RA (Fig. 4e and Supplementary Table 8). DE analysis of genes displaying opposite expression changes across the two trajectories identified 104 genes upregulated in the trajectory towards 2CLCs and downregulated towards differentiation (Fig. 4g). Furthermore, 750 genes were downregulated in the trajectory towards 2CLCs but upregulated towards differentiation. Altogether, 854 genes displayed transcriptional changes in response to RA across both trajectories. Gene list enrichment analysis revealed that GATA2 target genes (*P* value = 0.01089) were enriched in upregulated genes towards 2CLCs, in line with the known role of GATA2 in 2CLC induction^21^. By contrast, genes upregulated towards the differentiation trajectory were enriched in MAX targets (*P* = 4.952 × 10^−24^). Indeed, *Max* expression is downregulated exclusively across the 2CLC trajectory (Supplementary Table 8), suggesting a potential role for MAX in the distinctive response of ES cells to RA. Although the role of each of these pathways needs to be investigated, these data provide a basis for understanding the different responses elicited upon RA stimulation in ES cells.

### Early embryos display endogenous RA activity

The above results indicate that RA is a primary gatekeeper of 2CLC reprogramming. Accordingly, our scRNA-seq data reveal that components of the RA signaling pathway are expressed in 2CLCs (Fig. 5a). Whether such a signaling response is a ‘cell culture’ feature of 2CLCs or part of the regulatory network of totipotent cells in 2-cell embryos is unclear. Indeed, while RA plays a key role in cell differentiation at later developmental stages^22,35^, its receptors are expressed earlier^36^. We thus addressed whether the RA pathway is active in pre-implantation embryos. RNA-seq analysis revealed expression of proteins responsible for metabolizing retinol, RA transporters and the RA nuclear receptors prior to the blastocyst stage (Fig. 5b). RARγ displayed the highest expression levels at the late 2-cell stage (Fig. 5b), suggesting that RA may regulate gene expression in 2-cell embryos through RARγ. To test this, we asked if regulatory elements in 2-cell stage embryos contain RARE motifs. We interrogated ATAC-seq datasets^37^ and found that the RARγ motif is enriched in accessible regions in early stages compared to the ICM (Fig. 5c). The enrichment in RARE motifs was observed in 2-cell and 8-cell stage embryos, suggesting that RA activity may be important during several stages of pre-implantation embryogenesis.

Next, we addressed whether the embryos display RA activity. First, we examined the localization of the nuclear RA importers, which translocate to the nucleus to mediate RA signaling^24^. Because CRABP2 is the RA donor for RARs and FABP5 for RXRs, we focused on CRABP2 and found that its mRNA is maternally deposited (Fig. 5b). Immunostaining revealed nuclear localization of CRABP2 from the 2-cell stage onwards, but cytoplasmic in zygotes (Fig. 5d). This change in localization suggests that RA signaling may be activated at the 2-cell stage. Second, we addressed whether embryos display RA-dependent RARE transcriptional activity by microinjecting the RARE-GFP reporter in a late 2-cell stage blastomere (Fig. 5e). We monitored embryos 42–44 h later to allow for detectable GFP fluorescence. We detected RARE activity in the large majority of microinjected embryos, based on GFP fluorescence (Fig. 5f,g). This activity was RARE-dependent, because GFP was undetectable in most embryos injected with the reporter lacking RARE (Fig. 5f,g). Note that the fact that we did not see GFP expression in all embryos is expected in this type of experiment due to potential mosaicism upon plasmid injection^38^. The number of embryos expressing GFP was similar in controls (DMSO) and with RA (Fig. 5g), indicating that early embryos have endogenous RA activity. Thus, the pre-implantation embryo displays endogenous RA activity and has the machinery to regulate RARE-driven transcription.

### Inhibiting RA activity compromises cleavage development

**Fig. 6:**
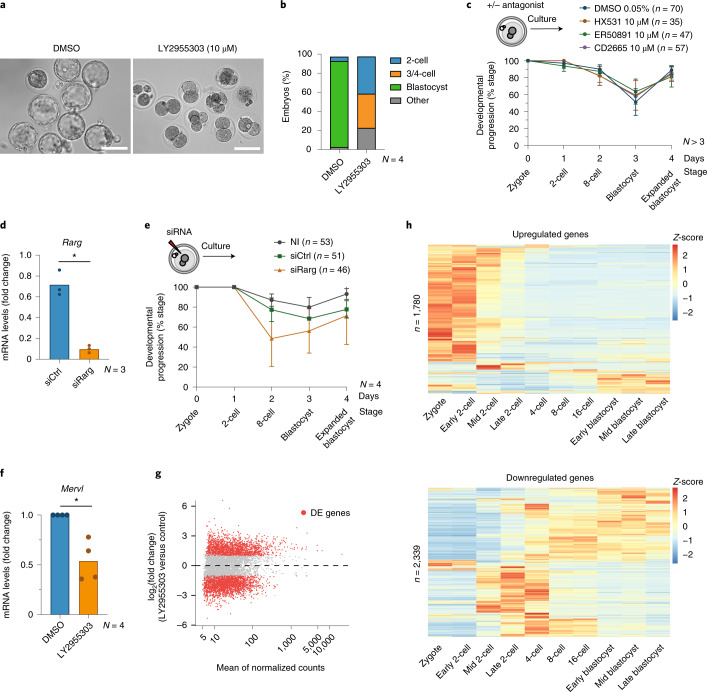
Perturbing RA signaling in the early mouse embryo affects developmental progression. **a**, Phase-contrast images of representative embryos treated with the RARγ antagonist LY2955303 or control DMSO. *N* = 4. Scale bars, 100 µm. **b**, Developmental progression (in percentage) of control (DMSO, *n* = 51) or embryos treated with the RARγ antagonist LY2955303 (*n* = 59 embryos). *N*, number of experimental replicates. **c**, Developmental progression of control (DMSO, *n* = 70) or embryos treated with the indicated antagonists against RXR (HX531, *n* = 35), RARα (ER50891, *n* = 47) and both RARβ and RARγ (CD2665, *n* = 57). Data are presented as mean values, and error bars represent s.d. *N*, number of independent replicates. **d**, qPCR analysis of *Rarg* in 2-cell stage embryos after siRNA for *Rarg* in zygotes. *N*, number of experimental replicates. *P* value calculated by two-sided Student’s *t*-test. **e**, Developmental progression of zygotes non-injected (*n* = 53) or microinjected with scramble siRNA (control; *n* = 51) or with siRNA against *Rarg* (*n* = 46). Data are presented as mean values, and error bars represent s.d. *N*, number of experimental replicates. **f**, qPCR analysis of *Mervl* transcripts after LY2955303 treatment. *N*, number of experimental replicates. *P* value calculated by two-sided Student’s *t*-test. **g**, MA plot showing differentially expressed genes in control (DMSO) 2-cell stage embryos versus LY2955303-treated embryos. Differential gene expression analysis was performed using DESeq2 (*P* values obtained by two-sided Wald test and corrected for multiple testing using the Benjamini and Hochberg method). Red color indicates log_2_FC > 1 or <−1; *P*_adj_ < 0.05. **h**, Heatmaps depicting the endogenous expression patterns of the up- and downregulated genes between embryos treated with LY2955303 versus control embryos at the late 2-cell stage. *Z*-score values are shown. RNA-seq datasets are from ref. ^52^ (Methods).

Finally, we investigated a potential role of RA signaling during the totipotency transition in embryos. To address whether RA signaling is important for pre-implantation development, we inhibited RAR signaling using a RARγ antagonist. We cultured zygotes with LY2955303 or the vehicle (DMSO). Control embryos formed blastocysts after three days (88%, *n* = 51). By contrast, inhibiting RARγ prevented developmental progression, with most embryos arrested at the 2-cell or 4-cell stage (78%, *n* = 59) (Fig. 6a,b). To investigate the potential involvement of other RA receptors, we treated embryos with three other antagonists against RXR homo- and heterodimers (HX531), RARα (ER50891) or both RARβ and RARγ (CD2665), but the latter with much lower affinity than LY2955303 (CD2665 Ki for RARγ is 100 times higher than LY2955303). None of these antagonists affected blastocyst formation, suggesting that only specific and robust chemical inhibition of RARγ affects developmental progression (Fig. 6c). To test this further we used siRNA against RARγ in zygotes, which led to a reduction of RARγ mRNA levels to ~8% of the controls (Fig. 6d). Knockdown of RARγ resulted in compromised developmental progression, with only ~60% of the embryos reaching the blastocyst stage (Fig. 6e). The milder phenotype observed with siRNA—as opposed to the RARγ antagonist—may be due to either incomplete protein knockdown and maternal deposition of RARγ, potential compensatory effects of other RA receptors upon RNAi, or LY2955303 potentially targeting other receptors. Unfortunately, our attempts to perform a RARγ western blot after siRNA were unsuccessful due to the low amount of material. Thus, although the RARγ antagonist treatment results in a much stronger phenotype, our siRNA results support a role for RARγ in regulating early developmental progression. However, we cannot formally exclude the possibility that other RA receptors may also be involved in RA signaling in early embryos.

Blocking ZGA with a general RNA PolII inhibitor results in most embryos arresting at the 2-cell stage^39^, similarly to the phenotype observed upon LY2955303 treatment. Thus, we next addressed if inhibiting RARγ affects ZGA by analyzing MERVL expression—a key ZGA marker—in embryos treated with LY2955303. qPCR revealed a striking reduction in MERVL transcripts in 2-cell embryos upon RARγ inhibition (Fig. 6f). These data suggest that RAR activity is necessary to ensure correct development prior to the 4-cell stage, presumably through regulation of ZGA. To address this, we performed RNA-seq^40^ in late 2-cell embryos upon LY2955303 treatment (Supplementary Fig. 7a,b). DE analysis revealed no significant differences between DMSO (vehicle) and potassium simplex optimized medium (KSOM) (control) embryos, so we performed all subsequent analyses against the DMSO group. Embryos grown with LY2955303 displayed a transcriptional program that differed from controls (Supplementary Fig. 7b). LY2955303 treatment led to significant changes in gene expression, with 1,780 upregulated and 2,339 downregulated genes (log_2_FC > 1 and log_2_FC < −1, respectively; *P*_adj_ < 0.05) (Fig. 6g and Supplementary Table 9). The majority of upregulated genes are normally highly expressed in zygotes and early 2-cell embryos (Fig. 6h), suggesting that LY2955303-treated embryos fail to progress into the transcriptional program of late 2-cell embryos. By contrast, most downregulated genes are highly expressed at the late 2-cell stage, which demarcates ZGA (Fig. 6h). Thus, chemical inhibition of RA signaling results in a failure to fully activate ZGA. Indeed, major ZGA genes were under-represented in the upregulated genes (*P* = 2.2 × 10^−16^, Fisher test) and over-represented in the downregulated genes (*P* = 2.723 × 10^−11^, Fisher test). Repetitive element expression was also affected by LY2955303, including downregulation of MERVL elements (MT2B2, MT2C_Mm and several MaLR) (Supplementary Table 9). Overall, our data suggest that RA signaling can control the ‘2-cell’ transcriptional program both in vitro, in cell culture, as well as in vivo, in mouse embryos.

## Discussion

Using a high-throughput, large-scale chemical screening, our work identifies a new regulatory pathway of 2CLC reprogramming and early mouse development. Consistent with our findings in 2CLCs, we identified a previously unappreciated activity of RA signaling at the earliest stages of embryogenesis. Thus, this work also helps to validate the use of 2CLCs as a model system for understanding the biology of the early embryo, enabling the discovery of a crucial signaling pathway at this stage of development.

Although several factors preventing the progression to a 2CLC state are known, much less is known about positive regulators promoting 2CLCs other than DUX^9,17,41^, DPPA2/4 (refs. ^18,19,42^) and miR-344 (ref. ^21^). Our data identify the RA signaling pathway as a core component of 2CLC identity and key regulator of 2CLC emergence. Previous work has shown that RA can increase the number of *Zscan4*^+^ cells in ES cell cultures^15,43^, which constitute around 5% of the ES cell population and are an intermediate cellular state between ES and 2CLCs^10^. In contrast to 2CLCs, RAR activity is not necessary for the emergence of the ZSCAN4^+^ population, although their numbers decrease when treated with a RAR inhibitor^15^. Together with previous work, our data support a model whereby RA induces both the ZSCAN4^+^ cells^43^ as well as the transition from the ZSCAN4^+^ state towards the 2CLC state. The identification of additional hits from our screening together with our findings on RA will enable the investigation of culture conditions to stably maintain 2CLCs. Our scRNA-seq dataset indicates that ES cells can undertake several paths in response to RA signaling and that 2CLCs are a clearly distinguishable, non-overlapping cell population, compared to early differentiating precursors. The fact that we did not detect additional cell populations between ES cells and 2CLCs in our scRNA-seq and velocity analyses may suggest that reprogramming towards the 2CLC state involves fast cellular transitions.

Whether the ability of ES cells to adopt distinct fates in response to RA signaling depends on the ability of RAR to target different genomic regions deserves further investigation. A possible mechanism whereby different doses of RA may cause different cellular responses could be the existence of different types of RA-responsive genes, for example, target genes with low versus high affinity for RARs binding, or with a different spacer length between the DR motifs. In such a scenario, a different output regarding gene expression results from different levels of transcription factor occupancy. This phenomenon has been documented for other nuclear receptors^44–46^, but has not been explored for RAR/RXR. Although pan-RAR antibodies have been used in the past^47^, the lack of antibodies specific for each RAR transcription factor has precluded this type of analysis. Notwithstanding, our observations that RAR motifs are significantly enriched in regulatory regions of 2CLCs and embryos at the 2- and 8-cell stages anticipates direct gene regulation by RA. Binding motifs for some transcription factors important for mouse development, such as *Nr5a2* and *Rarg*, do not show an enrichment in regulatory regions at the same stages in human pre-implantation embryos^48^. This suggests potential species-specific regulation, so a potential response to RA signaling of human induced pluripotent stem cells or ES cells will be exciting to investigate.

Identifying RA as a robust inducer of bona fide 2CLC reprogramming has allowed us to discover a new role for RA signaling in promoting the 2-cell stage program in vivo. In line with cell culture observations, chemical inhibition of RARγ results in developmental arrest, most probably due to a failure to fully trigger ZGA. Double compound mutants for RARα/RARγ are embryonic lethal at E7.5, and RARγ/RARβ double-deficient animals survive until birth^49,50^. In addition, although it is unclear whether RARγ^−/−^ females display reduced fertility, they can give rise to offspring^51^. Thus, although these studies did not reveal a pre-implantation phenotype when knocked out zygotically, their function during early development may have been obscured due to maternal inheritance and redundant activities. Indeed, the intricate functional redundancy of RAR and RXR, together with the compensatory effects by their different isoforms, renders their individual analysis complex^35^.

Altogether, our work sheds light into the regulatory networks underlying the reprogramming to a totipotent-like state in culture and suggests a previously unappreciated role for RA signaling at the earliest stages of mammalian embryogenesis.

## Methods

### Cell culture

Cells were grown in medium containing DMEM-GlutaMAX-I, 15% FBS, 0.1 mM 2-beta-mercaptoethanol, non-essential amino acids, penicillin and streptomycin and 2× LIF over gelatin-coated plates. Medium was supplemented with 2i (3 µM CHIR99021 and 1 µM PD0324901, Miltenyi Biotec) for maintenance and expansion. The 2i was removed 24 h before starting experiments.

### Flow cytometry

Before cytometry, cells were washed with PBS, trypsinized with trypsin-EDTA 0.1% and resuspended in 0.5% BSA PBS solution at 4 °C. Cells were kept on ice until sorting, performed using a BD BioSciences FACS Aria III. Analysis was done with FlowJo software (the gating strategy is shown in Supplementary Fig. 7c). For the RA effect on GFP^−^ cells experiment, the GFP^−^ gate was defined based on the fluorescence of wild-type (WT) ES cells and 2CLCs were removed before RA treatment. For scRNA-seq, treatments started at different timepoints so that all experimental conditions were collected at the same time. Samples were sorted to enrich the population in living single cells and library preparation was conducted immediately.

### Real-time polymerase chain reaction

Total RNA was extracted using phenol–chloroform extraction using TRIzol reagent (Invitrogen). Reverse transcription was performed with a First Strand cDNA synthesis kit (Roche) following the manufacturer’s instructions with random hexamers. Real-time PCR was performed with GoTaq qPCR Master Mix (Promega) on a LightCycler 96 Real-time PCR system (Roche). The relative expression level of each gene was normalized to *Rps28* and *Actb*. The primers used are listed in Supplementary Table 10. Data were plotted with GraphPad Prism.

### siRNA transfection

One day before transfection, 2i inhibitors were removed. siRNA transfection was performed using Lipofectamine RNAi MAX (Life Technologies). A total of 75,000 cells were transfected per condition and well in 24-well gelatin-coated plates, with a final siRNA concentration of 30 nM. Silenced Negative Control No.1 (Life Technologies) was used. The siRNAs are listed in Supplementary Table 9. The effect of siRNA silencing was examined three days after transfection and two days after RA treatment (qPCR primers are listed in Supplementary Table 11).

### Immunofluorescence

The *2C::turboGFP* cell line was cultured on gelatin-coated coverslips. At 48 h after RA treatment, cells were washed with PBS, fixed with 4% PFA for 10 min at room temperature and, after four washes with PBS, permeabilized with 0.3% Triton X-100 for 10 min at room temperature. After washing with PBS, primary antibodies were incubated overnight at 4 °C, followed by another three washes in PBS. The antibodies used were mouse turboGFP (TA140041, Origene) and rabbit Zscan4 (AB4340, EMD Millipore). Secondary antibodies were incubated for 1 h at room temperature. Mounting was done in Vectashield mounting medium (Vector Labs). Images were acquired using a Leica SP8 confocal microscope.

### Reporter cell lines

The *2C::tdTomato* and *2C::turboGFP/Zscan4::mCherry* lines have been previously described^10,16^. To generate *2C::turboGFP* reporter, ES cells were transfected with a plasmid containing a destabilized NLS-tagged turboGFP cassette under the regulation of *Mervl* LTR using Lipofecramine 2000. A single clone was selected from successfully transfected cells and has been fully characterized elsewhere (Nakatani et al., manuscript in preparation).

### Small-molecule screening

Plate and liquid handling was performed using an HTS platform system composed of a Sciclone G3 liquid handler from PerkinElmer with a Mitsubishi robotic arm (Mitsubishi Electric, RV-3S11), a MultiFlo dispenser (Biotek Instruments) as well as a Cytomat incubator (Thermo Fisher Scientific). Cell seeding and assays were performed in black 384-well CellCarrier plates (PerkinElmer, 6007558). The plates were coated with gelatin 0.1% for 20 min at 37 °C to facilitate better cell adherence. Cells were seeded in 384-well microplates with 10,000 cells per well. Image acquisition and image-based quantification was done using the Operetta/Harmony high-throughput imaging platform (PerkinElmer). *Z*′ factors were calculated according to the formula *Z*′ = 1 − (3(*θ*_p_ + *θ*_n_)/(*µ*_p_ − *µ*_n_)), where p is the positive control, n is the negative control, *θ* is the standard deviation and *µ* is the mean.

### Screening assay

*2C::turboGFP* ES cells were washed with 1× PBS, trypsinized and resuspended to a density of 90,909 cells ml^−1^ in cell culture medium. The cell suspension (10,000 cells per well; 110 µl per well) was dispensed into assay 384-well plates and incubated at 37 °C in 5% CO_2_. The same day, cells were treated either with compound (1 mM stock solution) dissolved in 100% dimethyl sulfoxide (DMSO) or DMSO alone, then 0.7 µl of compounds/DMSO were transferred to 110 µl cell culture medium per well to keep the final DMSO volume concentration below 0.7%. The positive control (10,000 cells per 110 µl) with 32 mM acetate and 0.7% DMSO was seeded separately after compound transfer in columns 23 and 24 of the 384-well assay plates. The cells were then incubated (37 °C, 5% CO_2_) for 48 h before fixation and antibody staining. Cells were permeabilized with PBS-Triton 0.3% for 5 min at room temperature (RT). After washing with PBS and blocking with PBS-BSA 1% for 1 h, primary anti-tbGFP antibody (TA140041) was added overnight at 4 °C. After washes with PBS, cells were incubated with Alexa488 anti-mouse secondary antibody, for 1 h at RT. After washes with PBS, cells were incubated with PBS-Hoechst 33342 (1 µg ml^−1^) for 15 min at RT. Cells were again washed with PBS. Finally, plates were recorded using the automated Operetta microscope using the ×20 NA objective for high-resolution images (PerkinElmer). For quantification, six images of each condition were recorded. This resulted in a cell number of ~100 cells of each condition in control wells with DMSO.

### Image analysis

Multiparametric image analysis was performed using Columbus high-content imaging and analysis software version 2.8.0 (PerkinElmer Life Sciences). Hoechst signal was used to detect cell nuclei using method C with the following parameters: common threshold (parameter determining the lower level of pixel intensity for the whole image that may belong to nuclei), 0.30; area (to tune the merging and splitting of nuclei during nuclei detection), >30 µm^2^; split factor (parameter influencing the decision of the computer of whether a large object is split into two or more smaller objects or not), 10; individual threshold (parameter determining the intensity threshold for each object individually), 0.2; contrast (parameter setting a lower threshold to the contrast of detected nuclei), 0.1. Next, the area of nuclei and the Hoechst intensity were determined and the nuclei were filtered by these properties (nucleus area >20 µm^2^ and <400 µm^2^; intensity > 100). For this subpopulation called ‘Nuclei selected’ the median intensity of the GFP signal was calculated and used to select the green cell population (intensity > 600). The percentage of the green cells was calculated. In addition, the whole image area was defined and the mean GFP signal was calculated to exclude wells with green fluorescent compounds (intensity < 400).

### Embryo collection and immunostaining

Experiments were carried out according to valid legislation and in compliance with the local government (Government of Upper Bavaria). Mice were bred in a 12-h light cycle. Housing conditions were according to ETS 123 guidelines: 20–24 °C and 45–65% humidity. Embryos were collected for immunostaining as described in ref. ^53^ from CD1 ~6-week-old females that were crossed with CD1 males upon natural matings. Embryos were fixed immediately after collection. The zona pellucida was removed with acid Tyrode’s solution (Sigma), and embryos were washed three times in PBS and fixed^54^. After permeabilization, embryos were washed three times in PBS-T (0.1% Tween in PBS), free aldehydes were removed by short incubation in NH_4_Cl (2.6 mg ml^−1^) and the embryos were washed twice in PBS-T. The embryos were blocked and incubated with anti-CRABP2 antibody, then washed three times in PBS-T, blocked and incubated with the corresponding secondary antibodies (A488-conjugated goat anti rabbit immunoglobulin-G). After washes in PBS-T and PBS, embryos were mounted in Vectashield with DAPI (Vector Laboratories) and imaged under a Leica SP8 inverted confocal microscope using a ×63 oil objective across 0.5-μm stacks. Blastocysts were mounted in three dimensions and imaged across a 1-μm stack.

### Microinjection and embryo manipulation

For the RARE::GFP reporter plasmid experiments, 2-cell-stage embryos were collected from 5–8-week-old F1 (CBAxC57BL/6J) females mated with F1 males 42–44 h post hCG injection. Ovulation was induced by injecting 10 IU pregnant mare serum gonadotropin (PMSG) (IDT Biologika) and human chorionic gonadotrophin (hCG) (MSD Animal Health) 48 h later. A single, random blastomere was microinjected with 1–2 pl of 20 ng μl^−1^ of the RARE plasmid or the plasmid without the RARE sequences. Dextran rhodamine (1 mg ml^−1^) was added as the microinjection control. Embryos were cultured in KSOM and monitored regularly. For RNAi, zygotes were collected from 5–8-week-old F1 (CBAxC57BL/6J) females mated with F1 males at 17–19 h post hCG injection and microinjected with 1–2 pl of 25 μM siRarg pool (Horizon Discovery M-04974-01-005) or siControl^10^. GFP mRNA (100 ng) was added as positive control for microinjection. Embryos were cultured in KSOM and monitored regularly. At 20 h post injection, some embryos were washed in PBS and frozen for qPCR. For the experiments with antagonists, zygotes were collected at 18 h post hCG injection and randomly allocated to the experimental groups, then cultured in the presence of 10 μM LY2955303, HX531, ER50891 or CD2665 (Tocris 3912, 2823 and 3800, respectively) in 0.05% DMSO or DMSO 0.05% in KSOM and scored daily for developmental progression. The data were plotted with GraphPad Prism.

### Embryo real-time qPCR

Total RNA was obtained from 20–25 2-cell embryos using the Arcutus PicoPure RNA isolation kit (Applied Biosystems 12204-01). Reverse transcription was performed with Superscript IV reverse transcriptase (Invitrogen 18090010) following the manufacturer’s instructions, with random hexamers. Real-time PCR was performed with Roche SYBR Green I Master Mix (04707516001) on a LightCycler 96 real-time PCR system (Roche). The relative expression level of each gene was normalized to *Gapdh* and *Actb*.

### Single embryo RNA-seq

Zygotes were collected at 18 h post hCG injection and cultured in the presence of 10 μM LY2955303 in 0.05% DMSO, 0.05% DMSO in KSOM or KSOM alone. Embryos were cultured until the late 2-cell stage (48 h post hCG), washed in PBS at 37 °C and flash-frozen in lysis buffer according to the Smart-Seq2 protocol. Libraries were verified using a 2100 Bioanalyzer (Agilent). Samples were paired-end sequenced at PE250 on an Illumina NovaSeq 6000 platform.

### Single-cell RNA-seq

Cells were collected after RA treatment and sorted for live single cells by FACS. Cell were then counted and tested for viability with an automated cell counter. Five thousand cells of the sample were then input into the 10X protocol. Gel bead-in-emulsion (GEM) generation, reverse transcription, cDNA amplification and library construction steps were performed according to the manufacturer’s instructions (Chromium Single Cell 3′ v3, 10X Genomics). Samples were run on an Illumina NovaSeq 6000 platform.

### Gene counting

Unique molecular identifier (UMI) counts were obtained using the kallisto (version 0.46.0) bustools (version 0.39.3) pipeline^55^. First, mouse transcriptome and genome (release 98) fasta and gtf files were downloaded from the Ensembl website, and 10X barcodes list version 3 was downloaded from the bustools website. We built an index file with the ‘kallisto index’ function with default parameters. Then, pseudoalignment was done using the ‘kallisto bus’ function with default parameters and the barcodes for 10X version 3. The BUS files were corrected for barcode errors with ‘bustools correct’ (default parameters), and a gene count matrix was obtained with ‘bustools count’ (default parameters). To estimate the *tbGFP* read counts, we used the *tbGFP* sequence available from GenBank (ID ASW25889.1) and followed the same procedure.

### Quality control and normalization

To remove barcodes corresponding to empty droplets, we used the ‘emptyDrops’ function from the R library ‘DropletUtils’ version 1.6.1 (ref. ^56^). For this, a lower threshold of 1,000 UMI counts per barcode was considered. Afterwards, quality control was performed using Python library ‘scanpy’ version 1.4.2 (ref. ^57^). Cells were filtered by fraction of mitochondrial reads and number of detected genes. Cells having more than 10% counts mapped to mitochondrial genes or fewer than 1,000 detected genes were removed (Supplementary Fig. 4). Then data from *tbGFP* expression were integrated and count tables from each timepoint were normalized separately using the R library ‘scran’ (version 1.14.0)^58^ as follows. First, the function ‘quickCluster’ was run, then size factors were calculated based on this clustering using the function ‘computeSumFactors’ with default parameters. Finally, the data were normalized using the computed size factors.

### Batch correction and regressing out of confounding effects

We performed batch correction on the data with LIF with the mutual nearest neighbors (MNN) method^59^ (function ‘mnn_correct’ from the ‘mnnpy’ library; https://github.com/chriscainx/mnnpy), using as input the log-transformed normalized counts of the genes that were in the list of top 3,000 highly variable genes (HVGs) at every timepoint, as done in ref. ^59^ (highly variable genes were identified with the function ‘highly_variable_genes’ in the scanpy library with the following parameters: min_disp=0.3, inplace=False, n_top_genes=3000). Afterwards, only genes with more than two counts in at least two cells were kept for further analysis and the data were scaled using the function ‘pp.scale’ from scanpy. On this batch-corrected data, the number of detected genes was regressed out using the scanpy function ‘regress_out’.

### Data visualization, clustering and diffusion maps

We used UMAP^60^ for data visualization (‘umap’ function in scanpy, with options n_components=2, min_dist=1). Leiden clustering was performed on the top 3,000 HVGs calculated across the whole dataset (with *k* = 15 and resolution = 0.4) using a correlation distance in the ‘pp.neighbors’ function from scanpy. To identify marker genes for a given cluster, first we found differentially expressed genes between that cluster and any other cluster (Wilcoxon’s rank sum test, false discovery rate (FDR) < 0.1, log_2_FC > 1), then genes were ranked according to their mean FDRs computed across all pairwise comparisons. To validate the differentiation state of the clusters suggested by the markers, the expression of some previously known relevant genes (*Rex1*, *Sox2*, *Nanog*, *Tcstv1*, *Zscan4a*, *Zscan4c*, *Zscan4d*, *Zscan4e*, *Gata6*, *Meis1*, *Sox17* and *Sox7*) was plotted on UMAP. Cells were aligned along a pseudotime trajectory using a diffusion map^61^, which was computed with the ‘diffmap’ function from the scanpy package on the first 20 principal components. We performed all differential gene expression analyses with Wilcoxon’s rank sum test, with an FDR threshold of 0.1 and log_2_FC threshold of 1.

### RNA velocity

To estimate RNA velocities^62^, we obtained loom files as described in the following. Fastq files were aligned using STAR (version 2.7.3a)^63^. Genome indices were generated using STAR --runMode genomeGenerate with default parameters. Then, alignment of reads was performed with the following options: --runThreadN 8 --outSAMunmapped Within. The resulting SAM files were converted to bam format and sorted using samtools^64^ (version 0.1.19-44428cd). Uniquely aligned reads from cells that passed the quality control were selected and distributed in separate bam files. We ran velocyto (version 0.17.17)^62^ with the option run-smartseq2 on bam files from cells corresponding to each timepoint to generate one loom file of spliced and unspliced counts per timepoint. On these loom files, we ran ‘scvelo’^65^ to perform RNA velocity analysis. This was done separately for the early timepoints (0 h, 2 h and 12 h) and the 48 h + LIF dataset. Second-order moments (steady-state levels) were calculated with the function ‘pp.moments’. These values were used for computing velocities using the function ‘tl.velocity’ with the following options: mode=‘stochastic’, min_r2=0.001. RNA velocity was plotted on a diffusion map colored by cluster with the function ‘pl.velocity_embedding_stream’ from scvelo.

### Cellular trajectory analysis

The trajectories analysis was performed in R (version 4.0.2) using the R package slingshot^66^ (version 1.6.1) on the 48 h dataset with the main clusters. As input for slingshot, we used the original main clusters (2, 3 and 5) and the diffusion map (function DiffusionMap from the R library destiny^67^ computed on the top 3,000 HVGs identified with the function FindVariableFeatures (with selection.method=‘vst’) from the R library Seurat. Data were normalized using the function NormalizeData (with parameter normalization.method equal to ‘LogNormalize’) from the R library Seurat^68^ (version 3.2.0). DE analysis was done with the R package tradeSeq^69^ (version 1.2.1). For detecting the DE genes along the two trajectories we used the function startVsEndTest. Identification of the genes that are most different between the two trajectories was performed with the function patternTest with parameters l2fc equal to log_2_(1.5) and nPoints equal to 50.

### Single-embryo RNA-seq analysis

Data quality was assessed with FastQC (version 0.11.7). Reads were processed with Trimmomatic (version .0.39) to remove Nextera adaptors and over-represented sequences. Reads were subsequently mapped to the mouse genome M25 (GRCm38.p6) and quantified using kallisto (version 0.44.0). Reads were imported into R (version 4.0.2) by the tximport package and the Scater and Single Cell Experiment packages were used to perform quality control tests by comparing library size, number of expressed genes and proportion of mitochondrial genes, for which the applied thresholds were 30,000 reads as the minimum for library size, 5,000 genes as minimum for the number of expressed genes and 20% as the maximum for the proportion of mitochondrial genes. Accordingly, one of the LY2955303 samples was removed as an ‘outlier’, because it did not pass the QC threshold (Supplementary Fig. 7a). Embryos with an average number of counts of ≥10 were kept for subsequent analysis. The average number of counts was calculated using the calculateAverage function from the scater package, where size-adjusted average count is defined by dividing each count by the size factor and taking the average across embryos. Principal component analysis was used to analyze the three groups of embryos (KSOM, DMSO or LY2955303) using log-transformed and library size-normalized counts using the top 3,650 high variable genes, which were calculated using modelGeneVar() and getTopHVGs() functions from the scran package. Differential gene expression analysis was performed using DESeq2 (version 1.28.1) with the threshold of an adjusted *P* value < 0.05 to select DE genes. Upregulated and downregulated DE genes from LY2955303 versus DMSO embryos with log_2_FC of >1 and <−1, respectively, were selected to show how they were expressed in WT embryos, based on RPKM values of published data^52^. RPKM values of the genes with non-zero counts were transformed to *Z*-scores to produce the relevant heatmaps. For repetitive elements analysis, trimmed reads were mapped to the primary assembly of the mouse genome M25 (GRCm38.p6) using STAR (version 2.7.6a) with the following parameters: --readFilesCommand zcat --outFilterType BySJout --outFilterMultimapNmax 100 --winAnchorMultimapNmax 200 --alignSJoverhangMin 8 --alignSJDBoverhangMin 1 --outFilterMismatchNmax 999 --alignIntronMin 20 --alignIntronMax 0 --alignMatesGapMax 0 --outSAMprimaryFlag AllBestScore --outMultimapperOrder Random --outSAMstrandField intronMotif --runRNGseed 13 --outSAMtype BAM Unsorted --quantMode GeneCounts --twopassMode Basic. Mapped reads to genes and TEs were counted using TEtranscripts (v.2.1.4), where the used GTF file for TE annotations was mm10_rmsk_TE.gtf. Finally, DE analysis was performed as described above using the count table generated from TEtranscripts. The list of ‘major’ ZGA genes has already been published^70^.

### Assay for transposase-accessible chromatin sequencing analysis and transcription factor binding site enrichment analysis

ATAC-seq data from 2CLC and ES cells^30^ (GSE75751) was downloaded, reads were trimmed using trimmomatic (version 0.38) with parameters 3:30:8:1:true LEADING:10 TRAILING:10 SLIDINGWINDOW:5:10 MINLEN:30. The output was aligned to the mm10 (vM21 GRCm38.p6) mouse genome from GENCODE, using bowtie2 with the parameters --dovetail --no-discordant --no-mixed -X 1500. BAM files were cleaned keeping the uniquely mapped reads using the samtools functions fixmate, sort and view -q 14. Peaks were called using macs2 v2.1.2.20181002 --bdg -q 0.01 -SPMR --keep-dup all --call-summits. The ATAC-seq data from mouse embryos^37^ (GSE66390) were preprocessed and aligned as above. Peak-calling was also done with macs2, with parameters --bdg -q 0.01 --nomodel --nolambda --keep-dup, all as reported by the authors of that study. The transcription factor binding site enrichment analysis was done using the software Analysis of Motif Enrichment (AME) from the MEME suite v5.0.5, using Fisher’s exact test to assess the relative enrichment and --kmer 1. The binding motif matrices used for the scanning were downloaded from JASPAR. 2CLC and ES cell RNA-seq (GSE75751) reads were trimmed in the same way as just described. The output reads were pseudoaligned with kallisto v0.44.0, using the mm10 (vM21 GRCm38.p6) mouse transcriptome available in GENCODE. Counts were normalized as RPKM. The RNA-seq data from mouse embryos were from GSE66390 and were processed following the same pipeline as for 2CLCs and ES cells RNA-seq.

### Statistical analyses

Statistical tests were performed keeping in mind the data distribution and the number of data points available. For all the qPCR analyses, because each replicate represents the mean expression level of the particular gene for thousands of cells, the data follow a normal distribution according to the central limit theorem. We thus applied the *t*-test (unpaired) for all statistically relevant comparisons. Across the manuscript, data on the percentage of 2CLCs in control conditions were gathered (*n* = 99) and a Shapiro–Wilk test was used to test if they were normally distributed. The test returned a significant *P* value, discarding a normal distribution. Therefore, a non-parametric test was used (Mann–Whitney, unpaired) to compare the 2CLC percentage between conditions whenever *N* ≥ 4. Additional details on sample sizes, in addition to the statistical tests conducted, are presented in the corresponding figure legends.

### Reporting Summary

Further information on research design is available in the Nature Research Reporting Summary linked to this Article.

## Online content

Any methods, additional references, Nature Research reporting summaries, source data, extended data, supplementary information, acknowledgements, peer review information; details of author contributions and competing interests; and statements of data and code availability are available at 10.1038/s41594-021-00590-w.

## Supplementary information

Supplementary Information Supplementary Figs. 1–7.

Reporting Summary

Supplementary Table 1 Top 20 marker genes for each cluster.

Supplementary Table 2 Genes upregulated in cluster A relative to all other clusters. Differential gene expression was found by Wilcoxon’s rank sum test and corrected for multiple testing using the Benjamini and Hochberg method.

Supplementary Table 3 Genes upregulated in cluster B relative to all other clusters. Differential gene expression was found by Wilcoxon’s rank sum test and corrected for multiple testing using the Benjamini and Hochberg method.

Supplementary Table 4 Genes upregulated in cluster C relative to all other clusters. Differential gene expression was found by Wilcoxon’s rank sum test and corrected for multiple testing using the Benjamini and Hochberg method.

Supplementary Table 5 Genes upregulated in cluster D relative to all other clusters. Differential gene expression was found by Wilcoxon’s rank sum test and corrected for multiple testing using the Benjamini and Hochberg method.

Supplementary Table 6 Genes upregulated in cluster E relative to all other clusters. Differential gene expression was found by Wilcoxon’s rank sum test and corrected for multiple testing using the Benjamini and Hochberg method.

Supplementary Table 7 Genes upregulated in cluster F relative to all other clusters. Differential gene expression was found by Wilcoxon’s rank sum test and corrected for multiple testing using the Benjamini and Hochberg method.

Supplementary Table 8 Differentially expressed genes along the trajectory towards 2CLCs and towards differentiation. Differential gene expression analysis was performed using tradeSeq.

Supplementary Table 9 Differentially expressed genes in DMSO and LY2955303-treated embryos. Differential gene expression analysis was performed using DESeq2.

Supplementary Table 10 Primers used in this study.

Supplementary Table 11 List of siRNAs used in this study.

## Data Availability

scRNA-seq data generated in this study are available under ArrayExpress accession no. E-MTAB-8869 and single-embryo RNA-seq data under accession no. E-MTAB-9940. All other data supporting the findings of this study are available from the corresponding author on reasonable request.

## Code availability

All scRNA-seq data were analyzed with standard programs and packages, as detailed in the Methods. Code is available on request.
