# Author Correction: Retinoic acid signaling is critical during the totipotency window in early mammalian development

**DOI:** 10.1038/s41594-022-00740-8

**Published:** 2022-02-14

**Authors:** Ane Iturbide, Mayra L. Ruiz Tejada Segura, Camille Noll, Kenji Schorpp, Ina Rothenaigner, Elias R. Ruiz-Morales, Gabriele Lubatti, Ahmed Agami, Kamyar Hadian, Antonio Scialdone, Maria-Elena Torres-Padilla

**Affiliations:** 1 Institute of Epigenetics and Stem Cells (IES), Helmholtz Zentrum München, Munich, Germany; 2 Institute of Functional Epigenetics (IFE), Helmholtz Zentrum München, Neuherberg, Germany; 3 Institute of Computational Biology (ICB), Helmholtz Zentrum München, Neuherberg, Germany; 4 Assay Development & Screening Platform, Institute of Molecular Toxicology & Pharmacology (TOXI), Helmholtz Zentrum München, Neuherberg, Germany; 5 Wellcome Sanger Institute, Wellcome Genome Campus, Hinxton, Cambridge, UK; 6 Faculty of Biology, Ludwig-Maximilians Universität, Munich, Germany

**Keywords:** Molecular biology, Developmental biology

Correction to: *Nature Structural & Molecular Biology* 10.1038/s41594-021-00590-w, published online 27 May 2021.

In the version of this article initially published, the surname of author Mayra L. Ruiz Tejada Segura was misspelled as Ruiz Tejeda Segura. The error has been corrected in the online version of the article.

