# Supplementary material for: Retinoic acid signaling is critical during the totipotency window in early mammalian development
Source: Nat Struct Mol Biol. 2021 May 27;28(6):521–32. doi: 10.1038/s41594-021-00590-w (PMC8195742; doi:10.1038/s41594-021-00590-w)
Supplement: Supplementary file 1 — Supplementary Figs. 1–7. [file 41594_2021_590_MOESM1_ESM.pdf]

nature portfolio

https://doi.org/10.1038/s41594-021-00590-w

Supplementary information

Retinoic acid signaling is critical during the totipotency window in early mammalian development

In the format provided by the authors and unedited

Supplementary Figure 1

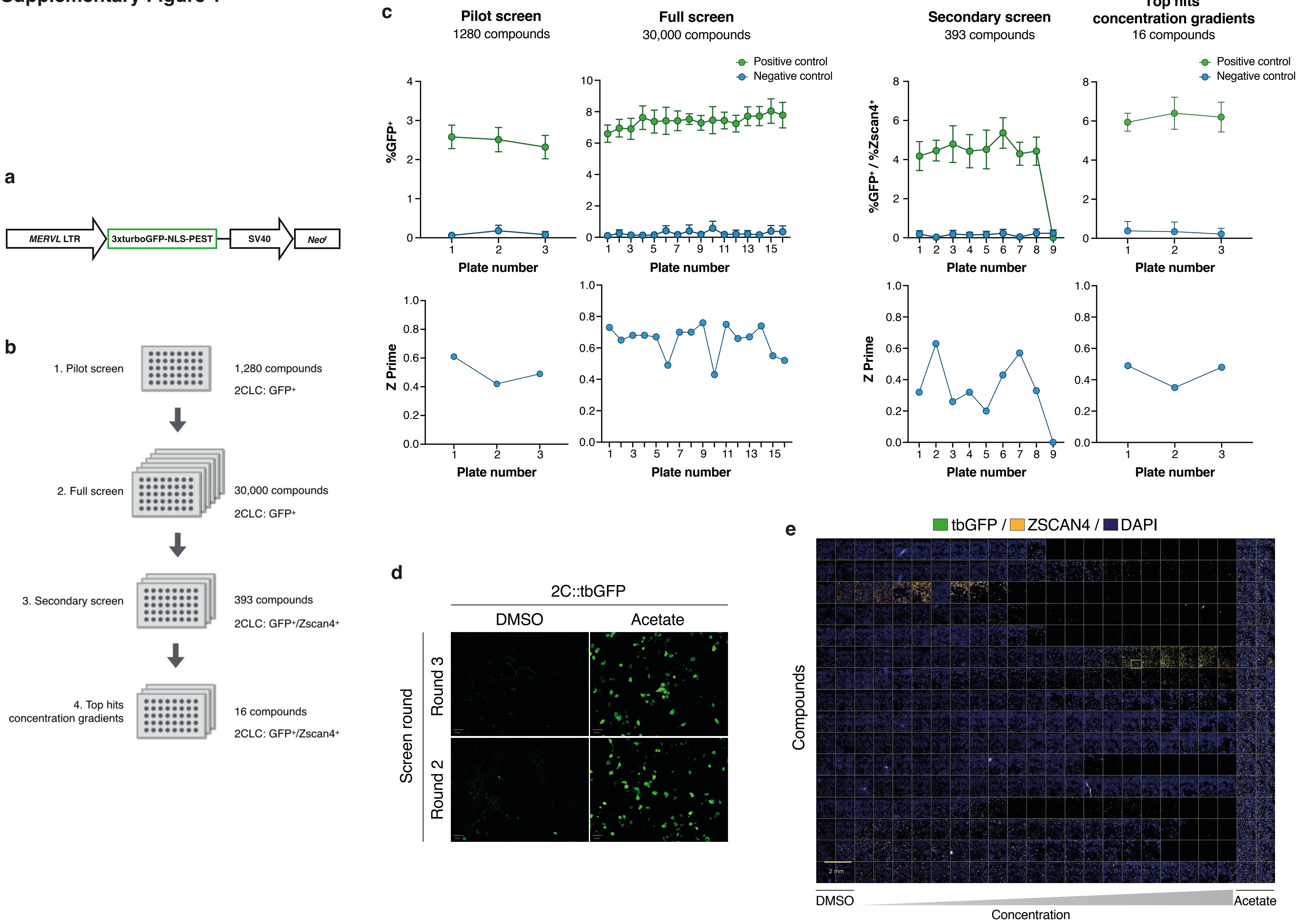

Supplementary Figure 1. Small molecule screen identifies retinoids as inducers of 2CLCs

a. Schematic representation of the 2C::reporter.

b. Design of the small molecule screen and the validation assays.

c. (Top) Quantification of GFP^+ and GFP^+/$Zscan4^+$ cells in control conditions (DMSO as negative control and Acetate as positive control) for plates across the pilot screen, one typical screening round with 30.000 diversity library compounds, secondary screen and final screen with top hits. Mean values ± s.d. from all control wells of a plate are shown. (Bottom) Z Prime values were calculated for each screening plate based on GFP^+ and GFP^+/$Zscan4^+$ quantification in control conditions (DMSO and Acetate).

d. Representative images of positive and negative controls in two different rounds of the full screen after immunofluorescence using a tbGFP antibody. Each plate of the screen counted with 32 wells for negative and positive controls.

e. Representative image of the final round of the screen (comprising 16 top hit compounds) after immunofluorescence with the indicated antibodies.

Supplementary Figure 2

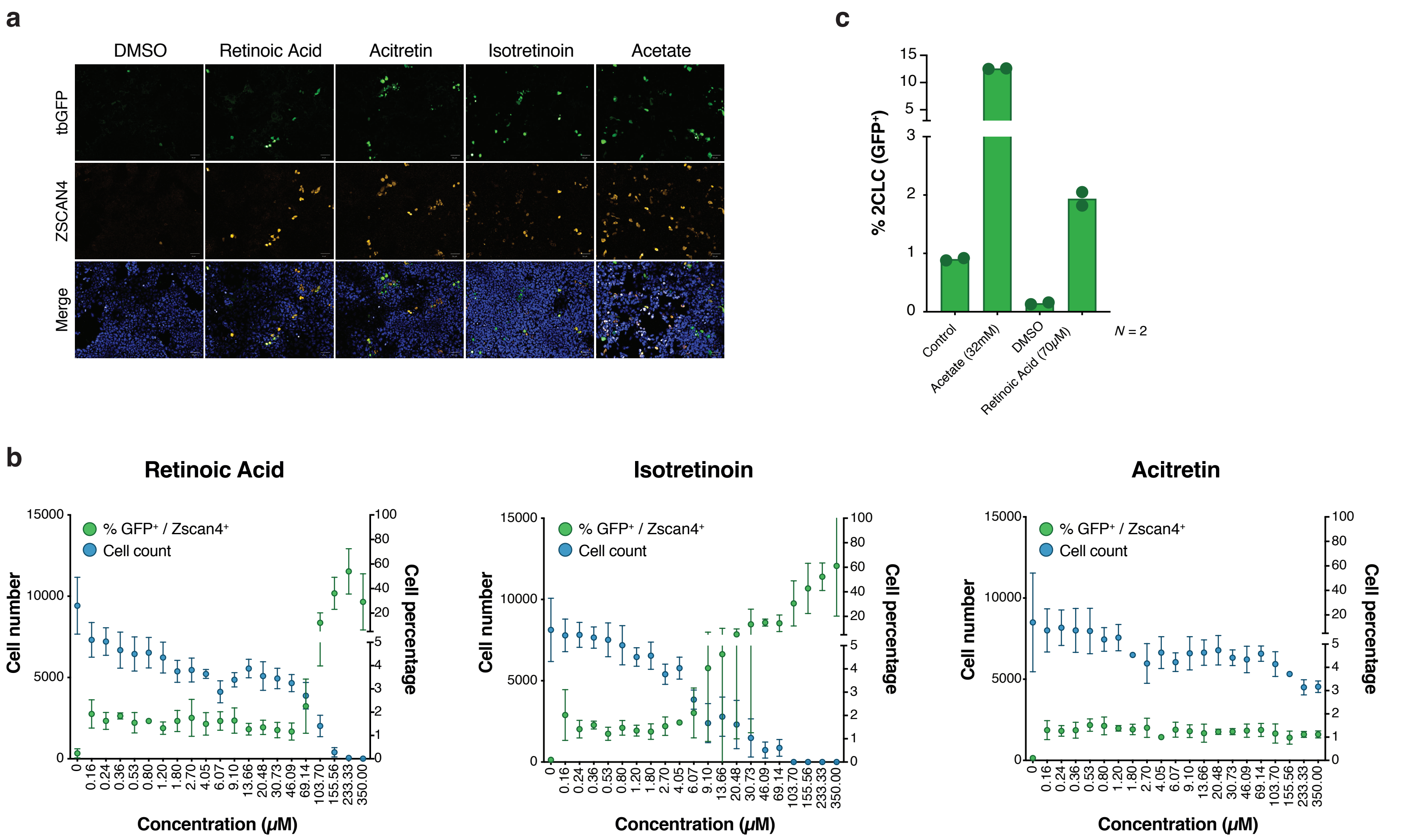

Supplementary Figure 2. Quality control of the small molecule screen identifying retinoids as 2CLC inducers

a. Representative images of ES cells with the retinoids identified as hits and the negative (DMSO) and positive (Acetate) controls from the last round of the screen (n=3 plate replicates). Scale bar, 50μm.

b. Quantification of 2CLCs, identified as double positive for GFP and ZSCAN4 (GFP^{+}/$Zscan4^{+}$), induced upon treatment with retinoids in a range of concentrations from the last round of the screen. Mean values ± s.d. from triplicate wells are shown. Total cell number is represented in blue.

c. Induction of 2CLCs (GFP^{+}) upon RA treatment measured by FACS. Control (no treatment), DMSO (RA vehicle) and Acetate (positive control) are shown. Mean of 2 replicates are shown. Each dot corresponds to the measurement of one replicate.

Supplementary Figure 3

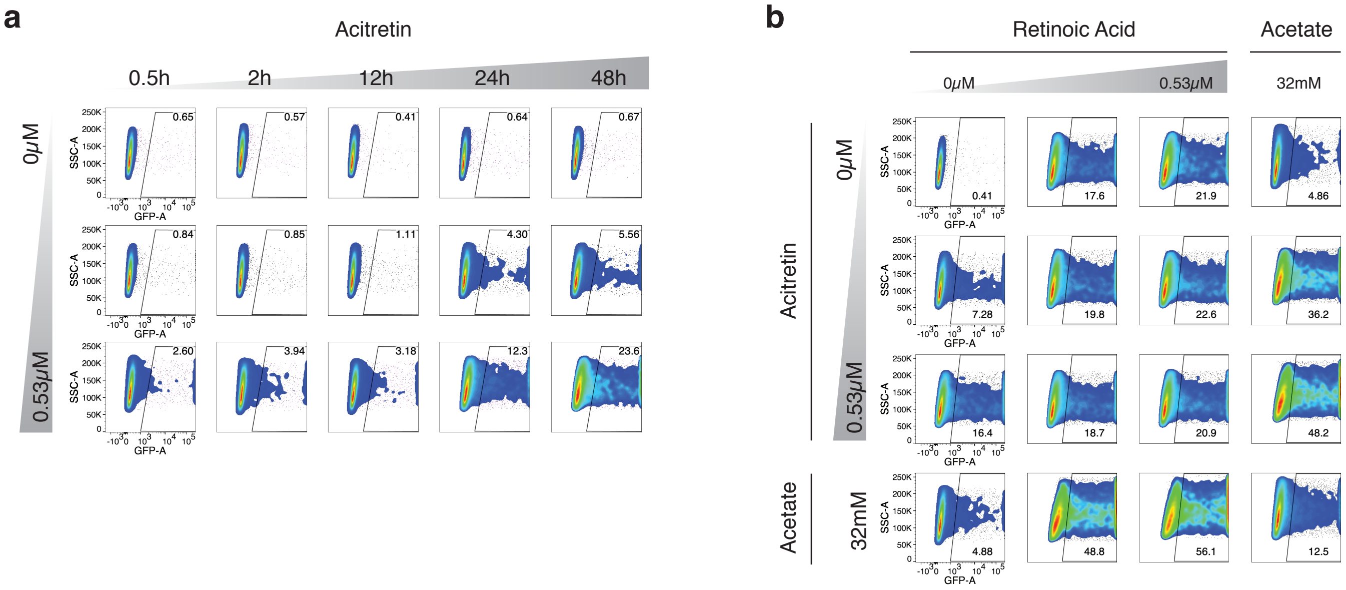

Supplementary Figure 3. Analysis of 2CLC induction by different retinoids and synergistic effects with Acetate

a. Representative scatter plots for experiment in Fig. 2a with Acitretin showing *2C::tbGFP* fluorescent measurements of individual cells as assayed by FACS.

b. Representative scatter plots for experiment shown in Fig. 2e showing *2C::tbGFP* fluorescent measurements of individual cells as assayed by FACS.

Supplementary Figure 4

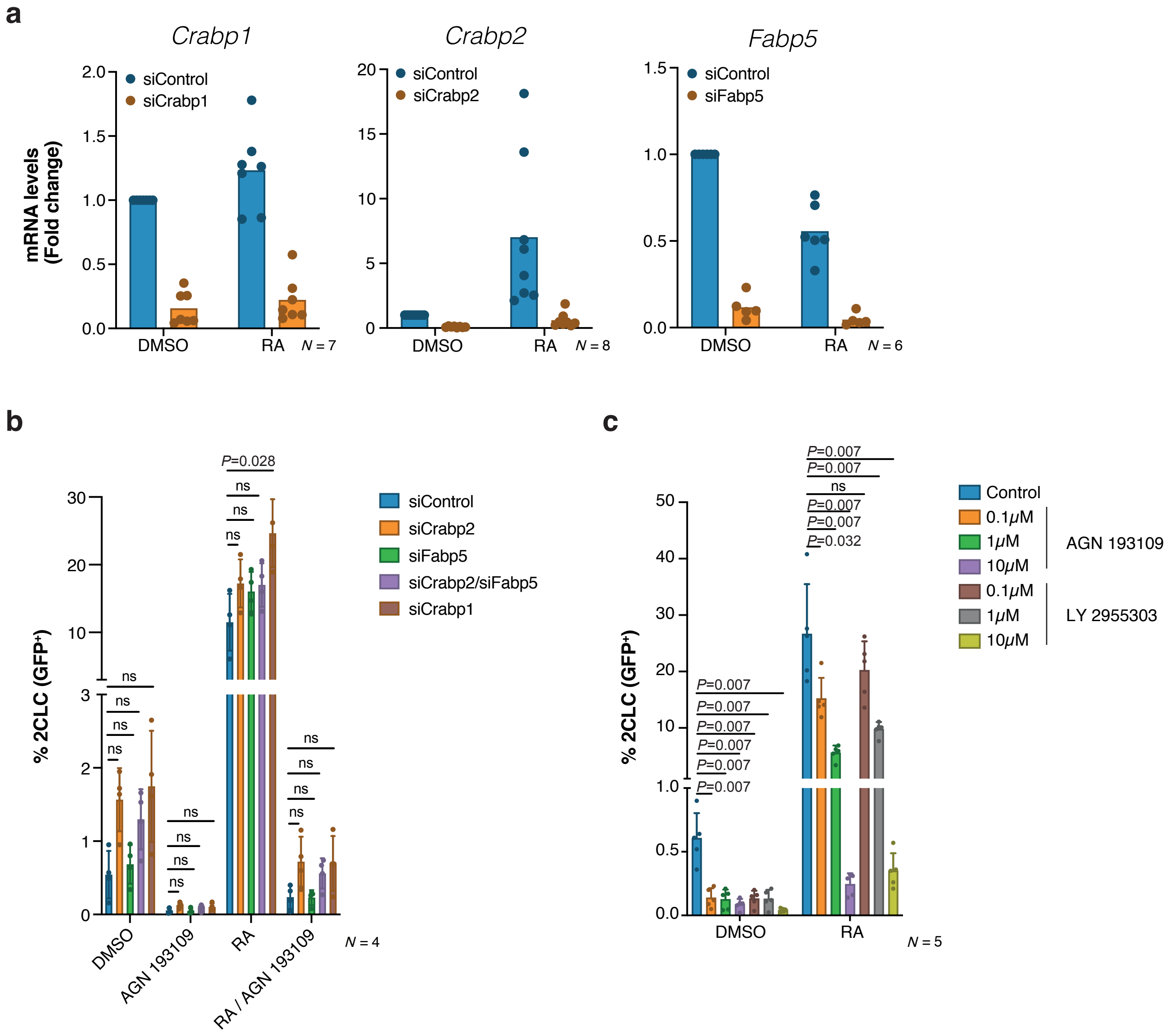

Supplementary Figure 4. Effect of perturbing RA pathway on 2CLC reprogramming.

a. RT-qPCR analysis of the indicated transcripts after siRNA for Crabp1, Crabp2 and Fabp5. Mean of the indicated replicates (represented by individual dots) is shown.

b. Induction of 2CLCs upon siRNA for Crabp1, Crabp2 and Fabp5 and RA and/or RAR antagonist AGN 193109 treatment. Scramble siRNA was used as control. Percentage of 2CLCs (GFP^+) quantified by FACS 48 hours after treatment. Mean ± s.d. of the indicated number of replicates is shown. *P*-value by paired two-sided Student's *t* test.

c. Induction of 2CLCs upon treatment with the RARγ antagonist LY2955303 or the RAR antagonist AGN193109. Percentage of 2CLCs (GFP^+) quantified by FACS 48 hours after treatment. Mean ± s.d. of the indicated number of replicates is shown. *P*-value by two-sided Mann-Whitney test.

Supplementary Figure 5

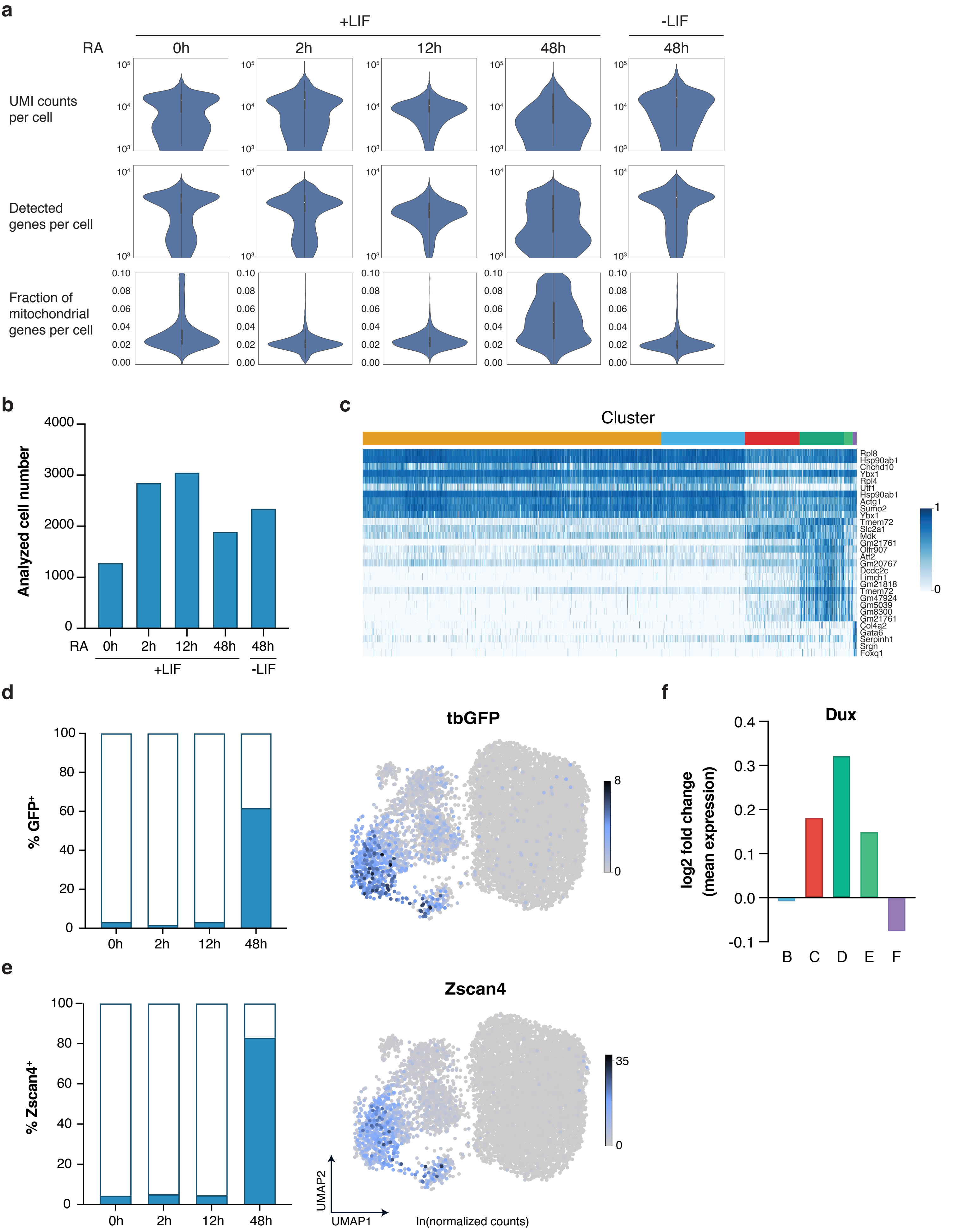

Supplementary Figure 5. Parameters of scRNA-seq analysis and quality controls.

a. The distribution of UMI counts, number of detected genes, and fraction of reads mapped to mitochondrial genes (rows from top to bottom) are shown with violin plots for cells in each condition (columns) after quality filtering.

b. Number of cells in each condition that passed quality control and were used for downstream analyses.

c. Heatmap illustrating the expression levels (normalized by the maximum) of the top 5 marker genes for each of the six clusters, indicated by the color bar at the top.

d. Percentage of cells where GFP is detected (left) and corresponding UMAP with cells colored by GFP expression (right) for cells grown in LIF and treated with RA during different times.

e. Percentage of cells where Zscan4 is detected (left) and corresponding UMAP with cells colored by Zscan4 expression (right) for cells grown in LIF and treated with RA during different times.

f. Bar plot showing the log2 fold change expression levels of Dux in different clusters. Fold change was calculated by dividing the mean of the ln(normalized counts) in each cluster by the mean of the ln(normalized counts) in cluster A.

Supplementary Figure 6

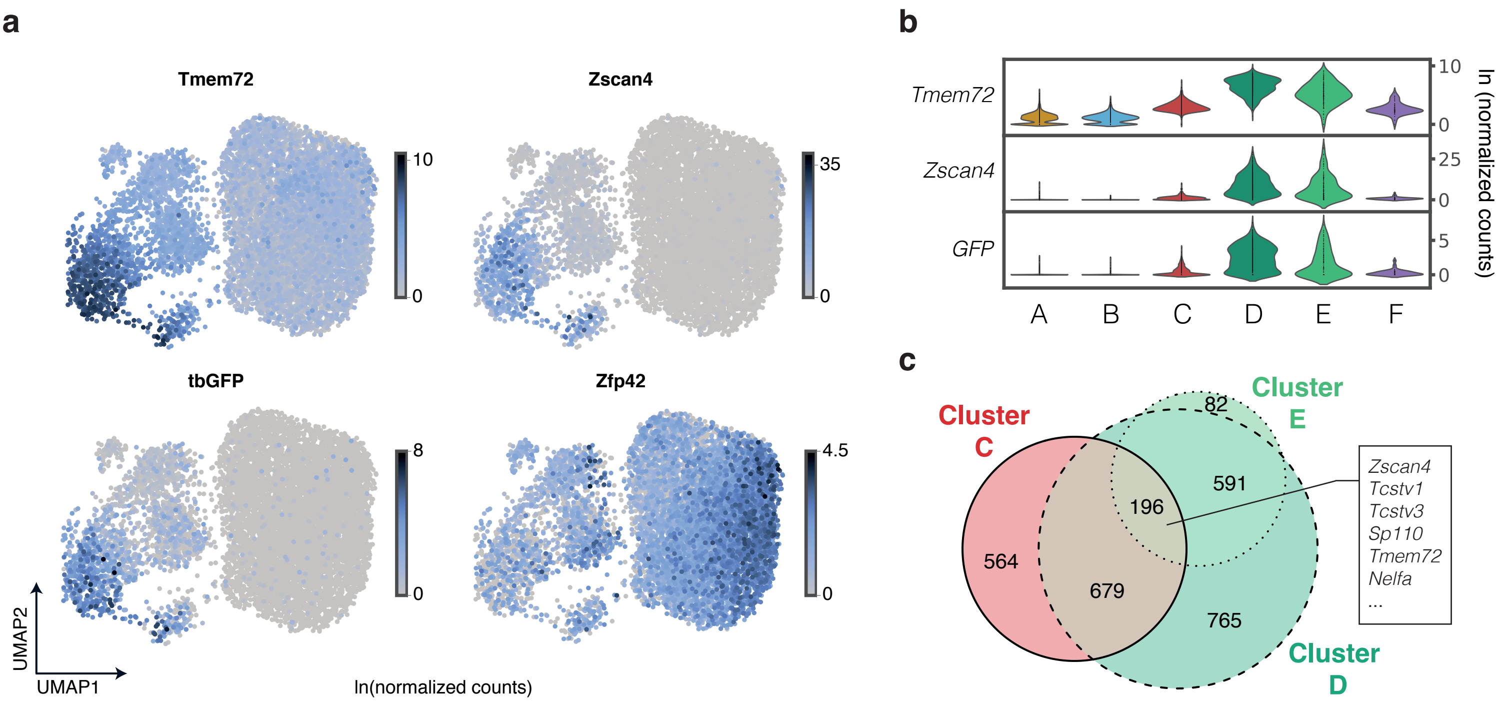

Supplementary Figure 6. Example of identification of new 2CLC markers

a. UMAP of all cells grown in LIF and Retinoic Acid, colored by expression levels of Tmem72 (top left), Zscan4 (top right); GFP (bottom left) and Zfp42/Rex1 (bottom right).

b. Violin plots showing the distribution of expression of 2CLCs marker genes Tmem72, Zscan4 and GFP (rows from top to bottom) per cluster (columns)**.**

c. Venn diagram comparing up-regulated genes (compared to cluster A) in Cluster C, Cluster D and Cluster E.

Supplementary Figure 7

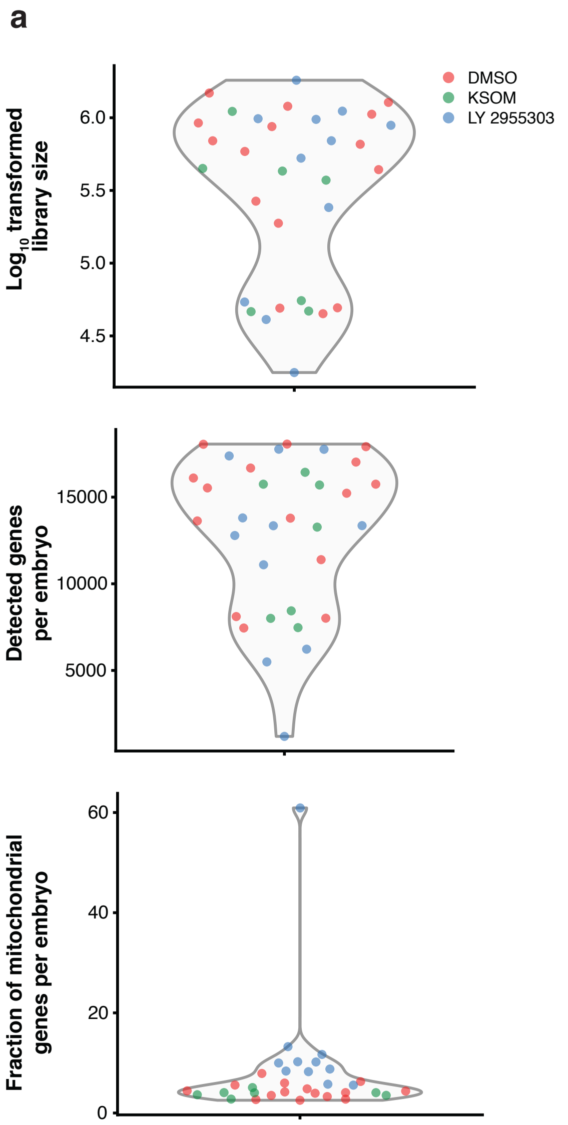

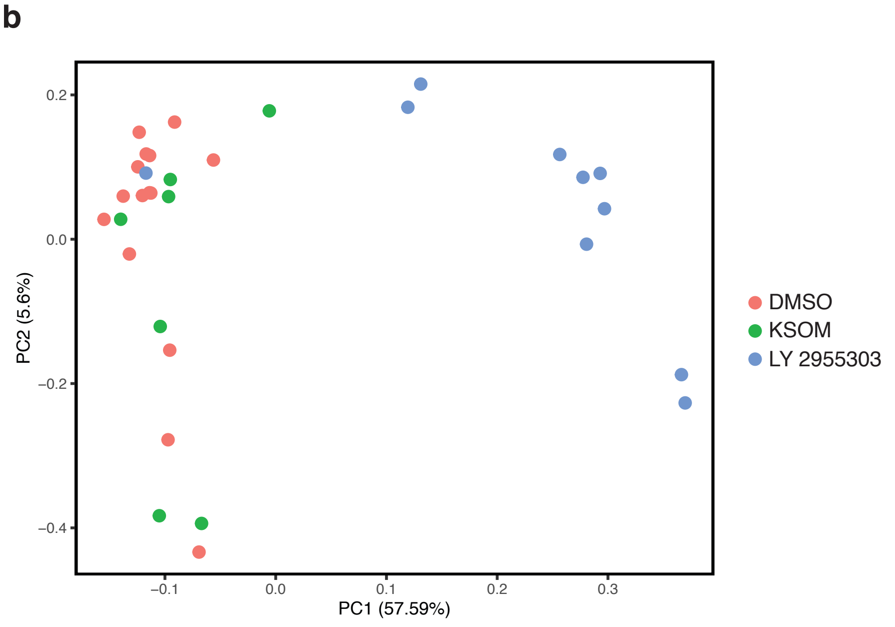

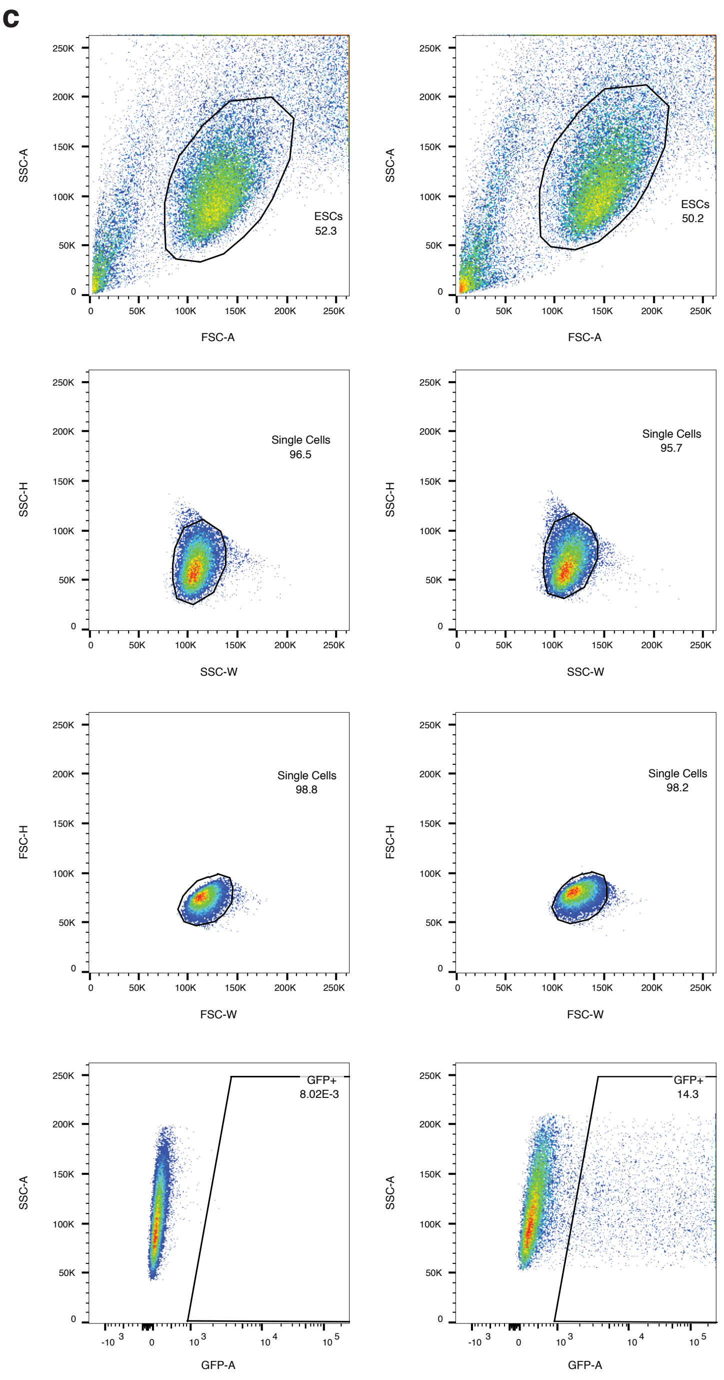

Supplementary Figure 7. Quality control of singe embryo RNA-seq analysis

a. Log10 transformed library size, number of detected genes per embryo, and fraction of reads mapped to mitochondrial genes per embryo are shown. Colour code corresponds to each experimental condition and each dot represents one embryo.

b. PCA of the single-embryo top HVG expression dataset. Each point corresponds to a single embryo, which is colored according to the experimental group it corresponds to.

c. Example of gating strategy used to quantify GFP^+ cells in FACS experiments. Left and right column corresponding to ES WT and 2C::tbGFP cells respectively.
