# Supplementary material for: Retinoic acid signaling is critical during the totipotency window in early mammalian development
Source: Nat Struct Mol Biol. 2021 May 27;28(6):521–32. doi: 10.1038/s41594-021-00590-w (PMC8195742; doi:10.1038/s41594-021-00590-w)
Supplement: Supplementary file 2 — Reporting Summary [file 41594_2021_590_MOESM2_ESM.pdf]

nature research

Corresponding author(s): Maria-Elena Torres-Padilla

Last updated by author(s): Apr 2, 2021

Reporting Summary

Nature Research wishes to improve the reproducibility of the work that we publish. This form provides structure for consistency and transparency in reporting. For further information on Nature Research policies, see Authors & Referees and the Editorial Policy Checklist.

Statistics

For all statistical analyses, confirm that the following items are present in the figure legend, table legend, main text, or Methods section.

n/a	Confirmed	
☐	☒	The exact sample size (*n*) for each experimental group/condition, given as a discrete number and unit of measurement
☐	☒	A statement on whether measurements were taken from distinct samples or whether the same sample was measured repeatedly
☐	☒	The statistical test(s) used AND whether they are one- or two-sided<br>*Only common tests should be described solely by name; describe more complex techniques in the Methods section.*
☒	☐	A description of all covariates tested
☐	☒	A description of any assumptions or corrections, such as tests of normality and adjustment for multiple comparisons
☐	☒	A full description of the statistical parameters including central tendency (e.g. means) or other basic estimates (e.g. regression coefficient) AND variation (e.g. standard deviation) or associated estimates of uncertainty (e.g. confidence intervals)
☐	☒	For null hypothesis testing, the test statistic (e.g. *F*, *t*, *r*) with confidence intervals, effect sizes, degrees of freedom and *P* value noted<br>*Give P values as exact values whenever suitable.*
☒	☐	For Bayesian analysis, information on the choice of priors and Markov chain Monte Carlo settings
☒	☐	For hierarchical and complex designs, identification of the appropriate level for tests and full reporting of outcomes
☒	☐	Estimates of effect sizes (e.g. Cohen's *d*, Pearson's *r*), indicating how they were calculated

Our web collection on statistics for biologists contains articles on many of the points above.

Software and code

Policy information about availability of computer code

Data collection	For small molecule screening, multiparametric image analysis was performed using Columbus high-content imaging and analysis software (version 2.8.0). For cell and embryo immunofluoresce image collections, Fiji (version 1.0) was used. FACS data was collected using Diva software from BD.
Data analysis	GraphPad Prism (version 8) and RStudio (version 1.1.383) were used for data analysis. Adobe Creative Suite was used for Figure preparation: Illustrator (CS6 version 16.0.0). The R programming language (versions R-3.6.3 and R-4.0.2) (https://www.R-project.org/) was widely used within the study for statistical analysis and data plotting, all custom code is avilable on request. For FACS experiments, data was analyzed using FlowJo (version 10).<br><br>For single cell RNAseq analysis, UMI counts were obtained using the kallisto (version 0.46.0) – Bustools (version 0.39.3) pipeline and the barcodes for 10x version 3. For quality control and normalization, R libraries DropletUtils (version 1.6.1) and scran (version 1.14.0) and Python library scanpy (version 1.4.256) were used. Data visualization was done using Leiden algorithm for clustering and plotting using UMAP with Python library scanpy (version 1.4.256). For RNA velocity, alignment was done with STAR (version 2.7.3a) and analysis with velocyto (version 0.17.17) and scvelo (version 0.1.24).<br><br>For single embryo RNAseq analysis, data quality was checked using FastQC (version 0.11.7), reads were processed with Trimmomatic (version 0.39) and quantified using kallisto (0.44.0). Reads were imported into R (version 4.0.2) by tximport package (version 1.16.1) and then Scater (version 1.16.2) and Single Cell Experiment (version 1.10.1) packages were used to perform quality control tests. Differential gene expression analysis was performed using DESeq2 (version 1.28.1). For repetitive elements analysis, trimmed reads were mapped using STAR (version 2.7.6a), mapped reads to genes and TEs were counted using TEtranscripts (version 2.1.4).<br><br>For ATAC-seq analysis, reads were trimmed using trimmomatic (version 0.38) and aligned with bowtie2. Peaks were called using macs2 (version 2.1.2.20181002). The transcription factor binding site enrichment analysis was done using MEME suite (version 5.0.5).

For manuscripts utilizing custom algorithms or software that are central to the research but not yet described in published literature, software must be made available to editors/reviewers. We strongly encourage code deposition in a community repository (e.g. GitHub). See the Nature Research guidelines for submitting code & software for further information.

Data

Policy information about availability of data

All manuscripts must include a data availability statement. This statement should provide the following information, where applicable:

- Accession codes, unique identifiers, or web links for publicly available datasets
- A list of figures that have associated raw data
- A description of any restrictions on data availability

All scRNAseq data are available at the ArrayExpress accession E-MTAB-8869.
Single cell embryo RNAseq data are available at the ArrayExpress accession E-MTAB-9940.

Previously published datasets re-analysed here are available under accession codes GSE75751 and GSE66390 (ATAC-seq) ; E-MTAB-2684 and GSE66390 (RNA-seq).

Field-specific reporting

Please select the one below that is the best fit for your research. If you are not sure, read the appropriate sections before making your selection.

☒ Life sciences ☐ Behavioural & social sciences ☐ Ecological, evolutionary & environmental sciences

For a reference copy of the document with all sections, see nature.com/documents/nr-reporting-summary-flat.pdf

Life sciences study design

All studies must disclose on these points even when the disclosure is negative.

Sample size	Sample size was chosen in order to ensure that the data was consistent and reproducible. See Figures and Figure legends for each experiment.
Data exclusions	Data in single cell and embryo RNAseq that did not pass quality control were excluded. The criteria for exclusion in quality control was pre-established as follows.<br><br>For single cell RNA-seq, to remove barcodes corresponding to empty droplets, a lower threshold of 1000 UMI counts per barcode was considered. Afterwards, cells having more than 10% counts mapped to mitochondrial genes or less than 1,000 detected genes were removed.<br><br>For single embryo RNA-seq, applied quality control thresholds were 30,000 reads as minimum for library size, 5000 genes as minimum for number of expressed genes and 20% as maximum for proportion of mitochondrial genes.
Replication	All data was replicated at least twice and the total replicate number is indicated in the respective panel. All attempts at replication were successful as reported in the manuscript with the exception of Figure 5g. For this experiment, four independent experiments were performed (as indicated in the panel). In one of the replicates, injection of RARE::GFP construct in DMSO condition did not present GFP+ embryos. This is represented in the Figure.
Randomization	2C blastomere to be injected was selected randomly and embryos were allocated at random to experimental groups as stated in the Methods.
Blinding	No experiment presented a subjective data collection that would require blinding. Experimentors were not blinded during experimental group allocation, embryos were divided randomly between groups.

Reporting for specific materials, systems and methods

We require information from authors about some types of materials, experimental systems and methods used in many studies. Here, indicate whether each material, system or method listed is relevant to your study. If you are not sure if a list item applies to your research, read the appropriate section before selecting a response.

Materials & experimental systems

n/a	Involved in the study
☐	☒ Antibodies
☐	☒ Eukaryotic cell lines
☒	☐ Palaeontology
☐	☒ Animals and other organisms
☒	☐ Human research participants
☒	☐ Clinical data

Methods

n/a	Involved in the study
☒	☐ ChIP-seq
☐	☒ Flow cytometry
☒	☐ MRI-based neuroimaging

Antibodies

Antibodies used	Antibodies used were as follows: (dilutions): anti-turboGFP (TA140041, Origene), ZSCAN4 (AB4340, EMD Millipore)(1:1000),

	CRABP2 (TA349827, Origene)(1:300).<br><br>Secondary antibodies used were: A-11029, A32732, A32731. Dilutions: 1:1000 for cells, 1:500 for embryos.
Validation	Anti-turboGFP antibody was validated by FACS using ES WT cell line (Supplementary Figure 7c). Anti-ZSCAN4 antibody was validated using a Zscan4c::tdTomato reporter cell line in Rodriguez-Terrones, D., Nat Genet 50, 106–119 (2018). Anti-CRABP2 was validated by the manufacturer (https://www.origene.com/catalog/antibodies/primary-antibodies/ta349827/crabp2-rabbit-polyclonal-antibody).

Eukaryotic cell lines

Policy information about cell lines

Cell line source(s)	The 2C::tdTomato and 2C::turboGFP/Zscan4::mCherry cell lines were previously described (Ishiuchi, T. et al., Nat. Struct. Mol. Biol. 2015; Rodriguez-Terrones, D. et al., Nat. Genet. 2018).<br>To generate 2C::turboGFP reporter cell line, ES cells were transfected with a plasmid containing a destabilized NLS-tagged turboGFP cassette under the regulation of Mervl LTR using Lipofecramine 2000. A single clone was selected from successfully transfected cells and has been fully characterized elsewhere (Nakatani et al., submitted).
Authentication	The 2C::tdTomato and 2C::turboGFP/Zscan4::mCherry cell lines were characterized in Ishiuchi, T. et al., Nat. Struct. Mol. Biol. 2015; Rodriguez-Terrones, D. et al., Nat. Genet. 2018). 2C::turboGFP reporter cell line has been also characterized (Nakatani et al., submitted).
Mycoplasma contamination	All cell lines tested negative for mycoplasma contamination.
Commonly misidentified lines<br>(See ICLAC register)	No commercially misidentified cell lines were used.

Animals and other organisms

Policy information about studies involving animals; ARRIVE guidelines recommended for reporting animal research

Laboratory animals	Preimplantation mouse embryos were collected from 5-7 week old F1 (C57BL/6J x CBA/H) superovulated females crossed with F1 males (3-6 months old). Superovulation was induced by intraperitoneal injection of pregnant mare serum gonadotropin (PMSG, Intervet, 5 IU) and human chorionic gonadotropin (hCG, Intervet, 7.5 IU) 46-48 hours later.
Wild animals	This study did not use wild animals.
Field-collected samples	This study did not involve field-collected samples.
Ethics oversight	All experiments were approved by and performed under the compliance of the Government of Upper Bavaria.

Note that full information on the approval of the study protocol must also be provided in the manuscript.

Flow Cytometry

Plots

Confirm that:

- [x] The axis labels state the marker and fluorochrome used (e.g. CD4-FITC).
- [x] The axis scales are clearly visible. Include numbers along axes only for bottom left plot of group (a 'group' is an analysis of identical markers).
- [x] All plots are contour plots with outliers or pseudocolor plots.
- [x] A numerical value for number of cells or percentage (with statistics) is provided.

Methodology

Sample preparation	Mouse ES cells were washed with PBS, trypsinized and resuspendedin 3% BSA PBS.
Instrument	FACS Aria IIIu
Software	FlowJo v10
Cell population abundance	Whenever cell numbers were not an issue, fluorescence was verified after sorting and was usually 95 - 100%. Downstream experiments always confirmed a very high degree of sorting purity.
Gating strategy	Stringent gatings were always used, leaving a significant gap in between negative/positive populations.

- [x] Tick this box to confirm that a figure exemplifying the gating strategy is provided in the Supplementary Information.
